# Investigation mechanisms of action and resistance of *Edwardsiella ictaluri* to trans-cinnamaldehyde

**DOI:** 10.1371/journal.pone.0340053

**Published:** 2026-01-07

**Authors:** Hossam Abdelhamed, Munshi Mustafiz Riman, Mark A. Arick, Ali Sobhy Dawood, Basant Gomaa, Monzur Chowdhury, Larry A. Hanson, Attila Karsi

**Affiliations:** 1 Department of Comparative Biomedical Sciences, College of Veterinary Medicine, Mississippi State University, Mississippi State, Mississippi, United States of America; 2 Institute for Genomics, Biocomputing and Biotechnology, Mississippi State University, Mississippi State, Mississippi, United States of America; CU Faculty of Veterinary Medicine: Cairo University Faculty of Veterinary Medicine, EGYPT

## Abstract

The rise of multidrug-resistant (MDR) pathogens in aquaculture poses a significant threat to food safety and public health by facilitating the transfer of resistance across the food chain, underscoring the need for sustainable, non-antibiotic control measures. Trans-cinnamaldehyde (TC), a phytochemical with antimicrobial activity, is a promising alternative. While previous publications have established the antibacterial efficacy of TC against *Edwardsiella ictaluri*, the agent of enteric septicemia of catfish, its mechanism of action and the potential for bacterial adaptation after prolonged exposure remain undefined. Here, we addressed these gaps by investigating the antibacterial mechanism, the adaptive response of *E. ictaluri* to TC, and the vaccine potential of TC-adapted strains, with relevance to One-Health. The minimum inhibitory concentration (MIC) of TC against *E. ictaluri* was 120 µg/mL, whereas 16 µg/mL was not inhibitory and was used for long-term adaptation. Serial passages for 30 and 60 days generated D30- and D60-adapted strains. Disk diffusion assays indicated reduced susceptibility to TC and florfenicol but increased susceptibility to sulfamethoxazole/trimethoprim (SXT) in adapted strains. Virulence assays in catfish fingerlings (three tanks containing 10 fish per tank) demonstrated that D30-TC and D60-TC strains were highly attenuated. Moreover, vaccination with these strains conferred significant protection against wild-type challenge (63.64% and 73.64% survival compared to 20% in controls). Quantitative proteomics revealed the upregulation of multidrug efflux pumps and stress adaptation proteins and the downregulation of central metabolic pathways during TC exposure. Long-term adaptation was characterized by metabolic reprogramming, including enhanced energy and carbohydrate metabolism and suppression of virulence-associated secretion systems. Comparative genomics of D30-TC and D60-TC strains revealed shared mutations in genes related to membrane biosynthesis, energy metabolism, stress response, and regulatory pathways. Together, these findings highlight the potential of TC as an antimicrobial and a potential source of live-attenuated vaccine, while revealing adaptive mechanisms that may inform future strategies for controlling *E. ictaluri* infections.

## Introduction

Pond-raised channel (*Ictalurus punctatus*) and channel x blue (*I. furcatus*) hybrid catfish dominate the aquaculture industry in the United States, accounting for $437 million in 2023 [1]. Infectious diseases remain a significant challenge for catfish producers, causing an estimated 45% of inventory losses, with approximately 60% of these losses attributed to single or mixed bacterial infections [2]. The primary bacterial pathogens impacting the catfish industry include *Edwardsiella ictaluri*, which causes enteric septicemia of catfish (ESC); *Edwardsiella piscicida*, which causes edwardsiellosis; *Flavobacterium covae*, the etiological agent of columnaris disease; and virulent strains of *Aeromonas hydrophila* responsible for motile *Aeromonas septicemia* (MAS) [3–7]. Reflecting these industry-wide challenges, diagnostic surveillance in the Mississippi Delta documented 179 cases of ESC in 2022 (39.7% of all submissions) and 187 in 2023 (34.2% of all submissions), underscoring persistent ESC activity in recent years among producer samples [8]. Similarly, extension reports from Alabama identify ESC as one of the most frequently diagnosed bacterial diseases in commercial catfish ponds, underscoring its continued impact across production regions [9].

The most common practices for controlling mortality attributed to infectious diseases on catfish farms are the administration of medicated feeds and the implementation of feed restrictions [10,11]. While these strategies can be effective, they present several limitations. Medicated feeds are relatively costly, and their efficacy is significantly reduced once an outbreak has progressed to the point where fish stop feeding due to inappetence [12]. Besides, outbreaks of ESC have also been reported across catfish farms in the southern United States in recent years [13,14]. Moreover, the emergence of plasmid-mediated antimicrobial resistance (AMR) has been documented in *E. ictaluri*, raising concerns about the long-term sustainability of current treatment strategies [15–17]. Beyond farm-level impacts, the rise of MDR bacterial pathogens in aquaculture poses a broader One-Health and food safety concern. The widespread occurrence of MDR phenotypes among aquaculture bacterial isolates poses a significant risk of transferring resistance genes to human pathogens through environmental and food-chain exposure [18,19]. This underscores the urgent need for potent, safe antimicrobial alternatives and sustainable disease management strategies that reduce antibiotic use and minimize selective pressure driving AMR.

Plant-based treatments have gained significant interest in disease management due to their diverse bioactive properties and potential as alternatives to conventional antimicrobials [20]. Among them, essential oils such as trans-cinnamaldehyde (TC) have been widely recognized for their antimicrobial activities [21,22]. TC has demonstrated inhibitory effects against a broad spectrum of pathogens, including *Salmonella enteritidis*, *Campylobacter jejuni*, drug-resistant *Aeromonas hydrophila*, *Escherichia coli*, and methicillin-resistant *Staphylococcus aureus* [23–25]. Recent research has extended these findings to aquaculture pathogens, showing that TC exhibits potent antibacterial activity against *Flavobacterium columnare*, *A. hydrophila,* and *E. ictaluri* at concentrations of 20, 80, and 40 μg/ml [26]. Moreover, dietary supplementation with TC has been shown not only to reduce bacterial load but also to enhance the survival rates of infected catfish, which was associated with upregulated expression of immune-related genes, such as immunoglobulin M (IgM) and the activation of memory cell production, thereby strengthening both innate and adaptive immunity [27].

Despite promising findings, the specific mechanisms by which *E. ictaluri* responds to TC and the potential development of resistant strains against TC remain poorly understood. To promote the rational use of TC as an alternative to medicated feeds, it is crucial to gain a deeper understanding of its antimicrobial properties and mode of action. Previous research has demonstrated that antimicrobial-induced stress responses can impact cellular processes, including disruption of key metabolic pathways [28], damage to cytoplasmic and outer membranes [5], and alterations in membrane fatty acid composition during bacterial growth [29]. In addition, several phytochemicals function as efflux-pump inhibitors that suppress AcrAB-TolC/NorA-like systems and potentiate co-administered antibiotics, emphasizing the need to monitor efflux activity and gene expression when defining TC’s mode of action [30,31]. However, no prior study has systematically mapped and delineated *E. ictaluri*’s response to a phytochemical across molecular layers. This knowledge gap limits evidence-based deployment of TC in aquaculture and highlights the need for a comprehensive approach to fully understand TC’s antimicrobial activity and resistance development in this critical fish pathogen.

While the emergence of bacterial resistance to natural antimicrobials has received less attention compared to conventional antibiotics, it is becoming an emerging concern. Understanding how pathogens like *E. ictaluri* adapt to or resist the antimicrobial effects of TC is essential to evaluating its long-term effectiveness and sustainability in aquaculture. Potential resistance mechanisms may involve upregulation of efflux pumps, activation of stress response pathways, and genetic mutations affecting TC targets [32–34]. Understanding these mechanisms is critical for optimizing the use of TC as a sustainable antimicrobial agent in aquaculture and mitigating the risk of resistance development.

In this study, we uniquely integrate proteomic and transcriptomic approaches to investigate the molecular mechanisms underlying the action of TC against *E. ictaluri* and characterize its mode of action. We also generated and characterized TC-adapted strains to delineate potential resistance and adaptation pathways. To our knowledge, this is the first study to combine these comprehensive omics approaches with *in vivo* infection models in the context of *E. ictaluri,* providing an in-depth understanding of TC’s mode of action and resistance mechanisms. This approach highlights the novelty of connecting molecular changes with phenotypic attenuation of virulence under TC pressure, offering novel insights into bacterial adaptation pathways. By elucidating both the mechanism of action and adaptive responses of *E. ictaluri* to TC exposure, this work provides novel insights into its potential as an alternative agent for managing ESC. Furthermore, our findings contribute to a broader understanding of resistance development in aquaculture pathogens, supporting sustainable disease control strategies with minimal risk of resistance emergence.

## Materials and methods

### Bacterial strains, growth conditions, and MIC testing

*Edwardsiella ictaluri* 93–146 strain was grown in Brain Heart Infusion broth (BHI) and incubated at 30°C. A TC solution (95%) was purchased from Sigma-Aldrich (Burlington, MA, USA). The minimum inhibitory concentration (MIC) of TC against *E. ictaluri* was determined by the microdilution method [26]. Sterile 48-well polystyrene plates (Costar; Corning Incorporated) containing BHI broth were inoculated with 5 µL of an *E. ictaluri* 93–146 suspension standardized to 10^6^ colony-forming units per milliliter (CFU/mL). TC was added to the wells at concentrations ranging from 40 µg/mL to 160 µg/mL (0.04 µL/mL to 0.16 µL/mL, unit converted based on TC density ≈1.05 g/mL at ~95% purity). A negative control (BHI broth without TC) was also included. The plates were incubated at 30°C for 24 h, and bacterial growth was determined by measuring optical density at 600 nm (OD_600_). The experiment was repeated three times, and each concentration was tested in six replicates.

### Prolonged passage of *E. ictaluri* in TC

In this experiment, a TC concentration of 16 µg/mL (0.016 µL/mL), which permitted the growth of *E. ictaluri* after overnight incubation at 30°C (indicating no inhibitory effect), was selected to generate TC-adapted strains. Briefly, *E. ictaluri* was inoculated into BHI broth (five replicates) containing 16 µg/mL of TC and incubated overnight at 30°C. Following incubation, 10 µL of cultured *E. ictaluri* was transferred daily into fresh BHI broth with the same TC concentration for 60 consecutive days. In parallel, *E. ictaluri* 93–146 was passaged daily in BHI broth without TC to serve as a control. Strains exposed to TC for 30 and 60 days were designated D30-adapted and D60-adapted, respectively, while the control strain exposed to BHI without TC was defined as D30-BHI and D60-BHI strains. All strains were stored at −80°C for subsequent MIC testing and proteomic analysis.

### Determination of TC resistance in TC-adapted strains

The MICs of the D30-adapted and D60-adapted *E. ictaluri* strains were compared to those of the wild-type (non-adapted) strain and the BHI-adapted control strains. A disc diffusion assay was performed to further evaluate the susceptibility of the adapted D30-TC and D60-TC strains to TC, florfenicol (30 μg), and sulfamethoxazole/trimethoprim (SXT, 1.25/23.75 µg). Bacterial colonies were suspended in saline and adjusted to a turbidity equivalent to 0.5 McFarland standard. Subsequently, the cultures were spread onto Mueller-Hinton agar plates using a sterile cotton swab. Sterile paper discs were impregnated with 2 µL (≈1.05 g/mL) of TC and placed on the agar surface. Commercial antibiotic discs were used for florfenicol and SXT. The plates were then incubated at 30°C, and the diameters of the zones of inhibition were recorded in centimeters (cm).

### Experimental challenge with D30-adapted and D60-adapted strains

All fish challenge experiments were conducted under a protocol approved by the Institutional Animal Care and Use Committee at Mississippi State University (IACUC-22–338). The approved protocol included humane endpoints, and fish meeting established morbidity criteria were immediately euthanized by immersion in tricaine methane sulfonate (MS-222). Criteria for euthanasia were loss of equilibrium, remaining on the water surface, lack of response to external stimuli, severe clinical signs of disease, or cessation of opercular movement. Some of the fish died during the study because of the experimentally induced systemic infection due to its rapid progression. All personnel involved in this experiment received IACUC-approved training in animal care and welfare from the University Laboratory Animal Veterinarian.

We evaluated the pathogenicity of D30-adapted and D60-adapted strains in catfish fingerlings using an immersion challenge model [35]. In brief, specific pathogen-free (SPF) fingerlings were transferred into challenge tanks (10 fish per tank) and supplied with a continuous flow of dechlorinated well water at a constant temperature of 28°C. Catfish were divided into six groups of three tanks: (1) a negative control group, (2) a positive control group infected with wild-type *E. ictaluri* 93–146, (3) a group challenged with the D30-adapted strain, (4) a group challenged with the D60-adapted strain, (5) a group challenged with the D30-BHI strain, and (6) a group challenged with the D60-BHI strain. For the immersion challenge, water volume in each tank was reduced to 10 liters, followed by the addition of 100 mL of the appropriate bacterial culture to achieve approximately 10^7^ CFU/mL water. After a one-hour exposure, normal water flow was restored. Fish were monitored daily for mortality over a 21-day period. Bacterial recovery from moribund and dead fish was performed by streaking anterior kidney tissue onto BHI agar to confirm the presence of *E. ictaluri*. At 21 days post-immunization, the vaccinated fish were challenged with the *E. ictaluri* 93–146 strain using the same immersion protocol to evaluate vaccine efficacy.

### Proteomic analysis

#### Sample preparation of adapted strains.

To investigate the proteomic response of *E. ictaluri* during adaptation to TC, a comparative proteomic analysis was performed on the D30-adapted, D60-adapted, and wild-type parental strains. The experiment was carried out with four biological replicates for each group. All bacterial strains were inoculated into BHI broth and incubated overnight at 30˚C with shaking. Bacterial pellets were harvested and washed with phosphate-buffered saline (PBS). Following established protocols [36], including protein extraction, quantification, and enzymatic digestion, to generate peptide samples for downstream analysis.

#### Sample preparation of sub-MIC exposed and control strains.

To further elucidate the antibacterial mechanism of TC*, E. ictaluri* 93–146 was cultured in BHI broth supplemented with two different sub-inhibitory concentrations of TC: 90 µg/mL (^3^/_4_ MIC) and 60 µg/mL (^2^/_4_ MIC) of TC. A control group was cultured in BHI broth without TC. All cultures were incubated at 30˚C with shaking for 18 hours. Four biological replicates from each group were used. After incubation, bacterial pellets from each group were collected for proteomic analysis.

#### DIA mass spectrometry.

Untargeted proteomics analysis was performed using data-independent acquisition (DIA) mass spectrometry in combination with a chromatogram library approach, following established protocols [36,37]. Protein samples were reduced, alkylated, and enzymatically digested using sequencing-grade modified porcine trypsin (Promega). The resulting tryptic peptides were separated using a reverse-phase XSelect CSH C18 2.5 µm resin column (150 x 0.075 mm) on an UltiMate 3000 RSLCnano system (Thermo Fisher Scientific). Peptides were eluted over a 60-minute linear gradient from 98:2–65:35 buffer A:B, followed by electrospray ionization at 2.2 kV. Mass spectrometric analysis was performed using an Orbitrap Exploris 480 mass spectrometer (Thermo Fisher Scientific). For chromatogram library generation, six gas-phase fractions were collected using 4 m/z DIA spectra, with precursor isolation windows set at 4 m/z at a resolution of 30,000. Full precursor scans were acquired across an m/z range of 496–602 at a resolution of 60,000. Wide-window DIA acquisitions were performed across the m/z range of 385–1015 using a resolution of 60,000. A total of 50 DIA spectra were acquired using a staggered window pattern, each encompassing a 12 m/z precursor isolation window at a resolution of 15,000.

Peptide identification was performed against *E. ictaluri* 93–146 proteome from UniProtKB, supplemented with common contaminants (cRAP) and trypsin sequences. A target–decoy strategy (q-value ≤ 0.01 at both peptide and protein levels) was used to control false discovery rates (FDR), with reversed decoy sequences appended for FDR estimation. Search parameters specified trypsin/P as the protease, allowing up to two missed cleavages, with carbamidomethylation of cysteine set as a fixed modification and methionine oxidation and N-terminal acetylation set as variable modifications. Precursor and fragment ion mass tolerances were aligned with the high-resolution Orbitrap DIA acquisition settings. The six gas-phase fractionation runs were employed to generate a comprehensive spectral library, improving peptide identification and quantification accuracy.

### Whole genomic sequencing

To investigate genetic adaptations associated with TC exposure, whole-genome sequencing was performed on five *E. ictaluri* strains (D30-adapted, D60-adapted, D30-BHI, D60-BHI, and non-adapted control). All strains were cultured in BHI broth without TC. Genomic DNA was extracted from each strain using a standard bacterial genomic DNA isolation protocol and submitted to Plasmidsaurus for hybrid whole-genome sequencing, which combined Oxford Nanopore Technology (ONT) long reads with Illumina short reads (2 × 150 bp short reads). Raw reads were adapter-trimmed and quality-filtered and mapped to the reference genome *E. ictaluri*. Coverage statistics showed ≥99.0% genome breadth at ≥10 × coverage for all samples, with mean depths of approximately 120× for Illumina and 60× for ONT. Single-nucleotide changes and small insertions/deletions were retained only if the site was well covered (at least 20× with Illumina and 10× with ONT). Variants in low complexity/repetitive regions or within 5 bp of another indel were filtered out. We reported larger structural changes only when reads split across the breakpoint and a clear change in read depth with at least five long ONT supporting the event and agreement between callers at ≥70% overlap. We considered a change linked to TC adaptation only if it appeared in the TC-adapted (D30-TC and D60-TC) and absent from the matched BHI controls and the non-adapted control.

### qRT-PCR analysis of genes with proteomic changes

Quantitative real-time PCR (qRT-PCR) was performed to validate proteomic responses under two experimental conditions: [1] the D60-TC-adapted strain was compared to an adapted control grown in BHI (no TC), and *E. ictaluri* wild-type exposed to ¾ MIC TC (90 µg/mL) was compared to control. For each condition, three independent cultures (n = 3) were grown to mid-log (OD600 ≈ 0.3–0.4), and total RNA was extracted with the FastRNA™ SPIN Kit for Microbes (on-column DNase I). After quality-checking, one microgram of RNA was reverse-transcribed with SuperScript™ using random hexamers. Representative genes were selected from proteomic hits involved in efflux/resistance, nucleotide biosynthesis, electron transport/energy metabolism, and virulence factors. Primers (90–150 bp) were designed with Primer–BLAST (S1 Table) and validated by melt curve analysis. Expression was normalized to 16S rRNA and fold change was calculated by the 2^^−ΔΔCq^ method; results are reported as mean ± SEM from n = 3 biological replicates (technical duplicates pooled).

### Statistical analysis

Proteins were considered significantly differentially expressed if they exhibited a false discovery rate (FDR)-adjusted *p*-value < 0.05 and a fold change greater than 1.5. Functional enrichment analysis of differentially expressed proteins (DEPs) was conducted using the STRING database, with particular emphasis on enriched biological processes and pathways. An FDR threshold of < 0.05 was used to determine statistical significance in enrichment results. Percent mortality was arcsine-transformed to meet the assumptions of normality and analyzed using analysis of variance (ANOVA) via PROC GLM. Differences in the zones of inhibition between strains were analyzed using one-way ANOVA followed by Tukey’s HSD post hoc test. Normality and homogeneity of variances were assessed prior to analysis. If assumptions were violated, the Kruskal-Wallis test with Dunn’s post hoc correction was applied. A *p*-value < 0.05 was considered statistically significant.

## Result

### Determination of MIC value and impact of TC dose on growth

**Fig 1:**
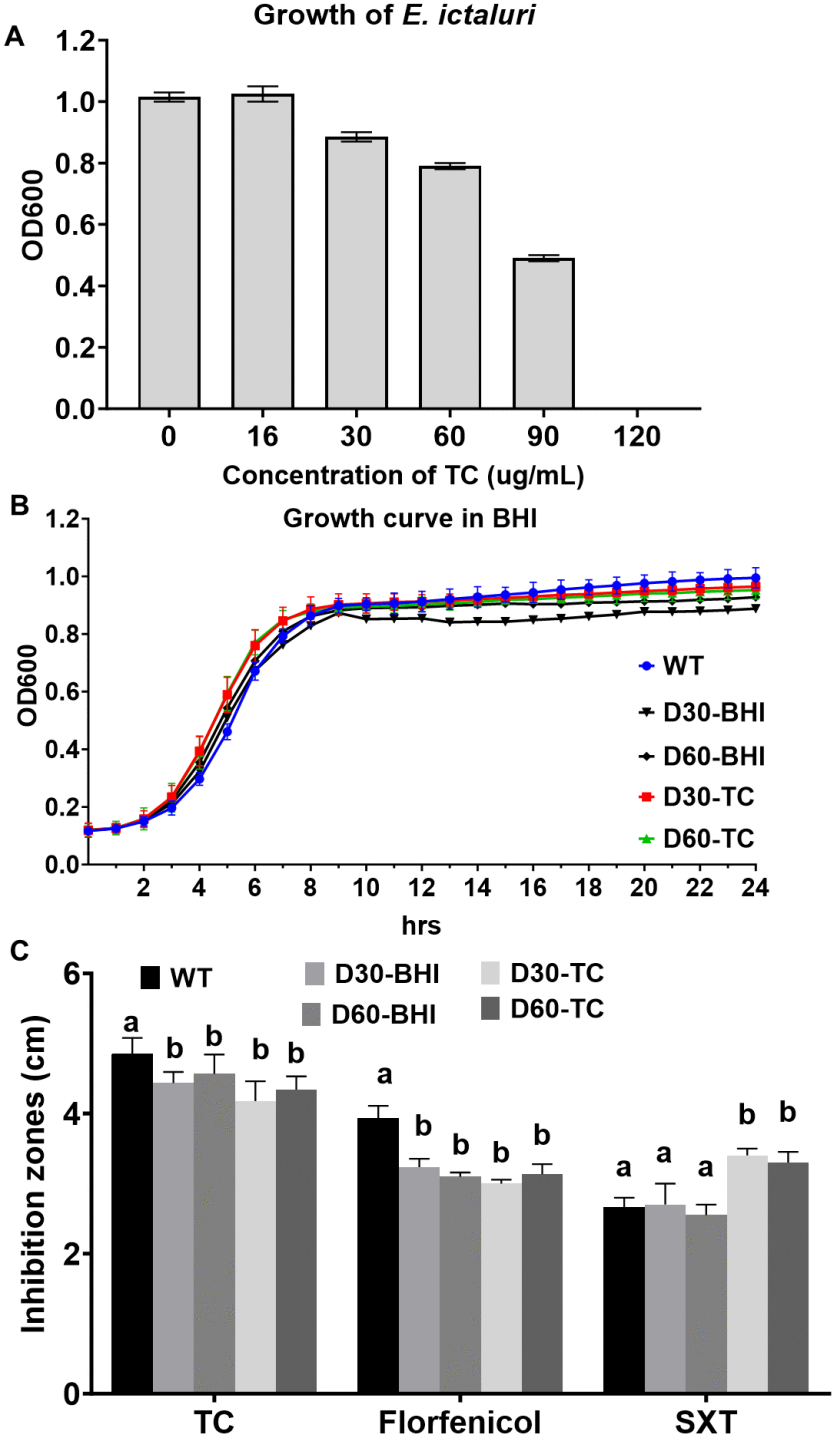
Growth response and susceptibility of *E. ictaluri* under TC exposure. **(A)** Growth response of *E. ictaluri* 93-146 to varying concentrations of TC. The MIC was determined as 120 µg/mL (0.12 µL/mL), with 90 µg/mL (0.09 µL/mL) and 60 µg/mL (0.06 µL/mL) selected as sub-MIC doses for subsequent proteomic studies, and 16 µg/mL (0.016 µL/mL) used for long-term exposure experiments. **(B)** Growth curves of *E. ictaluri* 93-146, D30, and D60 strains under standard conditions. **(C)** Inhibition zones of D30-TC and D60-TC adapted strains against TC, florfenicol, and SXT. Data represent mean inhibition zone diameters ± SEM from three independent experiments (n = 3). Different letters (a and b) indicate statistically significant differences (P < 0.05).

The MIC of TC against *E. ictaluri* 93–146 wild-type was determined to be 120 µg/mL (0.012%). As shown in Fig 1A, TC concentrations of 90 µg/mL (0.009%; ^3^/_4_ MIC), 60 µg/mL (0.006%; ^2^/_4_ MIC), and 30 µg/mL (0.003%; ^1^/_4_ MIC) significantly inhibited *E. ictaluri* growth compared to the untreated control (0 µg/mL). Accordingly, 90 µg/mL and 60 µg/mL were selected for proteomic experiments to investigate the mode of action and antibacterial activity of TC. Whereas 16 µg/mL (0.0016%; ^1^/_8_ MIC), which had no detectable effect on bacterial growth, was chosen for daily passaging of *E. ictaluri* over 60 consecutive days to study long-term adaptation to TC exposure.

### Long-term exposure of *E. ictaluri* to TC and cross-tolerance

D30-adapted and D60-adapted strains were generated through serial passaging of *E. ictaluri* 93–146 in BHI broth with 16 µg/mL TC for 30 and 60 days. These adapted strains exhibited growth behaviors similar to *E. ictaluri* and BHI-adapted control strains (Fig 1B), indicating that sub-MIC TC exposure did not impair bacterial growth.

To evaluate whether long-term exposure to TC influences *E. ictaluri* resistance to TC and alters its susceptibility to other antimicrobial agents, we measured the MIC and conducted disk diffusion assays (Fig 1C). No significant differences in MIC values against TC were observed for *E. ictaluri* 93–146 (non-adapted control) and D30-adapted and D60-adapted strains, indicating no substantial change in TC susceptibility. The inhibition zone assay demonstrated notable differences in antimicrobial susceptibility among the *E. ictaluri* wild-type and the adapted strains (Fig 1C). *E. ictaluri* wild-type exhibited inhibition zones measuring 5.0 cm for TC, 3.9 cm for florfenicol, and 2.7 cm for SXT. D30-BHI and D60-BHI control strains had slightly reduced zones for TC (4.4 cm and 4.6 cm) and florfenicol (3.2 cm and 3.1 cm), with similar or lower values for SXT (2.7 cm and 2.6 cm). Notably, the D30-TC and D60-TC strains demonstrated further reduced susceptibility to TC (4.2 cm and 4.6 cm) and florfenicol (3.0 cm and 3.1 cm) compared with both the wild-type and BHI-adapted strains, suggesting acquired adaptive mechanisms to tolerate TC and florfenicol effects. Interestingly, D30-TC and D60-TC strains exhibited increased susceptibility to SXT, as reflected by larger inhibition zones (3.4 cm and 3.3 cm) compared to both the wild-type and BHI-adapted controls. These results suggest that adaptation to TC may confer cross-tolerance to certain antibiotics while simultaneously increasing sensitivity to others.

### Long-term TC adaptation attenuates *E. ictaluri* virulence and confers vaccine potential in catfish

**Fig 2:**
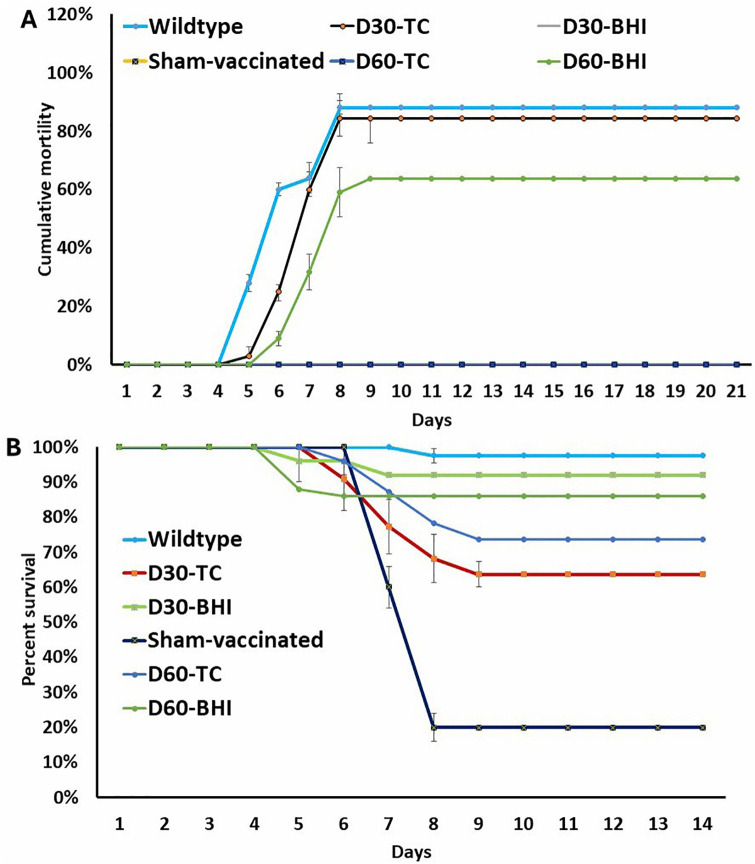
Virulence of *E. ictaluri* adapted strains and protective efficacy of adapted strain vaccines. **(A)** Cumulative mortality of catfish fingerlings challenged with *E. ictaluri* D30-TC, D60-TC, D30-BHI, D60-BHI, and *E. ictaluri* wild-type strains. **(B)** The percent survival of catfish vaccinated with D30-TC or D60-TC, followed by re-challenged with *E. ictaluri* wild-type at 21 days post-immunization.

The virulence and vaccine potential of the adapted strains were evaluated in catfish fingerlings using an immersion challenge model. Strikingly, no mortality occurred in the catfish fingerlings infected with D30-TC and D60-TC strains, whereas significantly higher mortality rates were recorded in those exposed to the wild-type, D30-BHI, and D60-BHI strains (88%, 84.37%, and 63.64%) (Fig 2A). At 21 days post-vaccination, survival rates were 63.64% and 73.64% in catfish vaccinated with the D30-TC and D60-TC strains compared to only 20% survival in the sham-vaccinated group (Fig 2B). These findings indicate that long-term TC adaptation attenuates *E. ictaluri* virulence and enhances its potential as a live-attenuated vaccine candidate.

### Proteomic alteration in *E. ictaluri* after TC exposure

**Fig 3:**
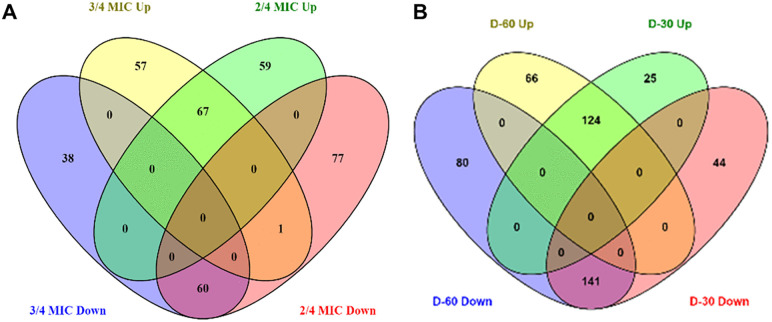
(A) A Venn diagram analysis using VENNY 2.1 was performed to visualize the overlap of enriched proteins in *E. ictaluri* exposed to ^2^/_4_ MIC and ¾ MIC of TC groups compared to the control. **(B)** Overlapping enriched proteins identified in D30- and D60-adapted strains compared to the control.

To investigate the mode of TC action against *E. ictaluri* 93–146, bacterial cultures were treated with two different concentrations of TC (^2^/_4_ and ¾ MIC) for 18 hours. Quantitative proteomics identified a total of 1420 distinct proteins across all samples. DEPs were defined by a fold-change threshold of ≥ 1.5 and a p-value of less than 0.05. In the ^2^/_4_ MIC group, 59 proteins were upregulated and 77 were downregulated compared with the control group, while in the ^3^/_4_ MIC group, 57 proteins were upregulated and 38 downregulated (Fig 3A and S2 Table). All the DEGs were subjected to Gene Ontology (GO) annotation and KEGG enrichment analysis to further characterize the functional impacts of TC exposure.

### Upregulation of efflux pumps and downregulation of central metabolic pathways in *E. ictaluri* exposed to ^2^/_4_ MIC and ^3^/_4_ MIC of TC

Comparative proteomics revealed 60 downregulated and 67 upregulated proteins that were commonly identified in both the ^2^/_4_ and ^3^/_4_ MIC of TC treatment groups compared to the control (Fig 3A). Enrichment analysis identified 8 proteins commonly upregulated and 37 proteins commonly downregulated proteins in both treatment groups (Table 1). Among the upregulated proteins were multidrug resistance protein MdtC, an efflux pump membrane protein, a TolC family outer membrane secretion protein, and a putative NAD(P)H nitroreductase, all associated with antimicrobial resistance and stress adaptation. Conversely, the consistently downregulated proteins were primarily involved in central metabolic pathways, including nucleotide biosynthesis (6 proteins; encompassing purine/pyrimidine synthesis and salvage), redox reactions and oxidoreductase activity (7 proteins), DNA repair and protein folding (5 proteins), amino acid and nitrogen metabolism (4 proteins), and stress response and envelope structure (5 proteins) (Table 1). These findings suggest that TC exposure was associated with stress-response mechanisms and inhibition of key metabolic functions.

**Table 1:** Commonly significant upregulated and downregulated proteins in the ^2^/_4_ MIC and ¾ MIC groups.

Locus tag	Description	Fold changes (^2^/_4_ MIC)	Fold changes (¾ MIC)
**Upregulation**			
**Efflux and resistance**			
NT01EI_1169	Multidrug resistance protein MdtC	3.01	3.39
NT01EI_3094	Efflux pump membrane protein, putative	2.00	3.01
NT01EI_1647	Putative NAD(P)H nitroreductase	3.46	2.93
NT01EI_0197	Type I secretion outer membrane protein, TolC family	1.84	2.81
NT01EI_2461	YcaO-like family protein	2.22	2.13
NT01EI_2772	Auxiliary transport protein, membrane fusion protein (MFP)	1.82	1.83
NT01EI_1098	Efflux pump membrane transporter	1.69	1.68
NT01EI_3093	Transcriptional regulator, MarR family	1.75	1.64
**Downregulation**			
**Nucleotide biosynthesis (purine/pyrimidine synthesis and salvage pathways)**			
NT01EI_3533	Carbamoyltransferase YgeW, putative	−17.6	−2.2
NT01EI_3530	Dihydropyrimidinase, putative	−7.5	−2.6
NT01EI_1174	Pyrimidine/purine nucleoside phosphorylase	−2.9	−2.4
NT01EI_0133	Uridine phosphorylase	−2.3	−2.4
NT01EI_2633	ribonucleoside-diphosphate reductase	−2.3	−2.0
NT01EI_0487	Anaerobic ribonucleoside-triphosphate reductase, putative	−1.9	−2.4
**Redox homeostasis and oxidoreductase activity**			
NT01EI_0686	Xanthine dehydrogenase, molybdenum-binding subunit	−8.0	−1.8
NT01EI_0684	Xanthine dehydrogenase, Fe-S subunit, putative	−2.3	−2.1
NT01EI_0929	Oxidoreductase molybdopterin binding domain protein	−2.4	−2.1
NT01EI_0971	Catalase-peroxidase	−1.71	−1.66
NT01EI_0252	Superoxide dismutase [Cu-Zn]	−1.9	−2.5
NT01EI_2672	NADH-quinone oxidoreductase subunit I	−1.7	−1.67
NT01EI_2864	Cytochrome D ubiquinol oxidase subunit 1, putative	−2.8	−2.1
**DNA repair and protein Folding**			
NT01EI_1290	Probable endonuclease 4	−1.7	−1.5
NT01EI_1249	DNA ligase	−1.8	−1.8
NT01EI_1196	Beta-barrel assembly-enhancing protease	−2.1	−2.0
NT01EI_3205	Chaperone protein ClpB	−1.7	−1.8
NT01EI_2491	ATP-dependent Clp protease ATP-binding subunit ClpA	−2.0	−1.9
**Amino acid and nitrogen metabolism**			
NT01EI_3529	Carbamate kinase	−7	−2
NT01EI_3532	Diaminopropionate ammonia-lyase, putative	−12	−2.5
NT01EI_2460	L-asparaginase, type 2, putative	−2.3	−1.5
NT01EI_2170	3-isopropylmalate dehydratase large subunit	−1.6	−2
**Metal and ion binding and storage**			
NT01EI_3531	M20/DapE family protein YgeY, putative	−11.4	−2.1
NT01EI_3523	Selenium metabolism protein SsnA, putative	−12	−1.6
NT01EI_3091	Ferritin-like domain protein	−3.5	−4.1
NT01EI_1879	Ferritin/DPS protein domain-containing protein	−2.4	−2.3
**Energy metabolism and electron transport**			
NT01EI_1217	Periplasmic nitrate reductase	−7.3	−3.5
NT01EI_2078	Fumarate hydratase class II	−1.7	−2
**Cofactor and vitamin biosynthesis**			
NT01EI_1057	6,7-dimethyl-8-ribityllumazine synthase	−2.16	−2.75
NT01EI_1056	Riboflavin biosynthesis protein RibD	−2.03	−1.96
**Carbohydrate metabolism**			
NT01EI_1736	Fructosamine kinase	−1.95	−1.97
NT01EI_0719	3-isopropylmalate dehydrogenase	−1.69	−2.60
NT01EI_2903	beta-N-acetylhexosaminidase	−1.57	−1.59
**Stress response and cell envelope**			
NT01EI_1637	PrkA serine protein kinase	−1.62	−1.78
NT01EI_0683	Phosphatidylethanolamine-binding protein	−1.52	−1.75
NT01EI_1415	Bifunctional polymyxin resistance protein ArnA	−1.51	−1.67
NT01EI_1943	Serine protease	−1.71	−1.57
**Hypothetical function**			
NT01EI_0900	Uncharacterized protein	−1.93	−6.23

Further enrichment analysis revealed consistent upregulation of protein clusters associated with RND (Resistance-Nodulation-Division) efflux pump, membrane fusion proteins, and efflux transmembrane transporter activity in both ^2^/_4_ and ^3^/_4_ MIC TC-treated groups (S3 Table). In contrast, protein clusters in purine metabolism, pyrimidine metabolism, and amidohydrolase-related functions were significantly downregulated in both the ^2^/_4_ MIC and ^3^/_4_ MIC groups compared to the control. GO analysis also showed a marked downregulation of proteins associated with catalytic activity in the ^2^/_4_ MIC (89 proteins) and ^3^/_4_ MIC (67 proteins) groups compared to the control.

### Proteomic profiling of *E. ictaluri* exposed to ^2^/_4_ and ^3^/_4_ MIC of TC

In the ^2^/_4_ MIC TC treatment group, enrichment analysis using the KEGG database revealed significant upregulation in histidine metabolism (5 proteins), amino acid metabolism (5 proteins), central metabolism and energy production (6 proteins), cofactor and vitamin biosynthesis (3 proteins), and sulfur metabolism and detoxification (1 protein) compared to the control group (Table 2). Conversely, oxidoreductase activity was significantly downregulated, with 27 proteins in redox processes showing decreased abundance relative to the control (Table 2).

**Table 2:** Most significant upregulated and downregulated proteins in the ^2^/_4_ MIC-treated *E. ictaluri* sample compared to the non-treated control group.

Locus tag	description	Fold changes	P.Value
**Upregulation**			
**Histidine metabolism**			
NT01EI_2570	Imidazole glycerol phosphate synthase subunit HisF	2.57	7.29E-05
NT01EI_2564	ATP phosphoribosyltransferase	2.06	8.40E-06
NT01EI_2567	Histidine biosynthesis bifunctional protein HisB	1.82	4.67E-05
NT01EI_2566	Histidinol-phosphate aminotransferase	1.77	3.86E-03
NT01EI_2565	Histidinol dehydrogenase	1.60	1.37E-03
**Amino acid metabolism**			
NT01EI_3900	Aspartate--ammonia ligase	3.36	2.99E-03
NT01EI_2124	Bifunctional protein PutA	2.39	9.99E-07
NT01EI_3846	Acetylglutamate kinase	2.04	5.13E-04
NT01EI_0916	Gamma-glutamyl phosphate reductase	1.66	6.26E-03
NT01EI_3106	Glutamate-1-semialdehyde 2,1-aminomutase	1.62	5.57E-04
**Carbohydrate metabolism and energy**			
NT01EI_0441	Fructose-1,6-bisphosphatase class 1	1.72	1.70E-03
NT01EI_3619	Phosphoglycolate phosphatase	2.00	8.16E-03
NT01EI_0759	Acetyltransferase component of pyruvate dehydrogenase	1.96	4.63E-06
NT01EI_2315	Isocitrate dehydrogenase [NADP]	1.74	4.99E-04
NT01EI_1608	6-phosphogluconolactonase	1.55	4.12E-04
NT01EI_0760	Dihydrolipoyl dehydrogenase	1.51	4.60E-04
**Cofactor and vitamin biosynthesis**			
NT01EI_2647	o-succinylbenzoate synthase	1.95	9.34E-03
NT01EI_0687	4-hydroxy-tetrahydrodipicolinate reductase	1.78	2.65E-05
NT01EI_2648	1,4-dihydroxy-2-naphthoyl-CoA synthase	1.73	7.08E-04
**Lipid metabolism**			
NT01EI_2718	Acetyl-coenzyme A carboxylase carboxyl transferase subunit beta	1.61	4.47E-03
**Sulfur metabolism and detoxification**			
NT01EI_3376	S-adenosylmethionine synthase	1.62	8.60E-04
NT01EI_3682	Thiosulfate sulfurtransferase GlpE	2.55	9.61E-04
**Downregulation**			
**Oxidoreductase activity (FDR = 0.0328)**			
NT01EI_0686	Xanthine dehydrogenase, molybdenum-binding subunit	−8.06	3.00E-10
NT01EI_1217	Periplasmic nitrate reductase	−7.26	2.00E-06
NT01EI_3522	Selenate reductase, FAD-binding subunit, putative	−5.24	8.35E-09
NT01EI_3091	Ferritin-like domain protein	−3.51	5.19E-06
NT01EI_0601	Thiol:disulfide interchange protein DsbL	−3.23	1.46E-03
NT01EI_0390	Fumarate reductase subunit C	−2.79	3.21E-04
NT01EI_2864	Cytochrome D ubiquinol oxidase subunit 1, putative	−2.75	3.98E-04
NT01EI_3369	D-erythrose-4-phosphate dehydrogenase	−2.48	1.97E-05
NT01EI_1879	Ferritin/DPS protein domain-containing protein	−2.43	4.51E-04
NT01EI_0929	Oxidoreductase molybdopterin binding domain	−2.41	6.04E-04
NT01EI_2633	ribonucleoside-diphosphate reductase	−2.33	6.49E-04
NT01EI_0684	Xanthine dehydrogenase, Fe-S subunit, putative	−2.28	3.24E-03
NT01EI_3524	Selenate reductase YgfK, putative	−2.20	2.34E-05
NT01EI_2464	Pyruvate formate-lyase-activating enzyme	−2.11	4.09E-03
NT01EI_1056	Riboflavin biosynthesis protein RibD	−2.03	4.92E-03
NT01EI_0392	Fumarate reductase flavoprotein subunit	−1.96	1.57E-05
NT01EI_0252	Superoxide dismutase [Cu-Zn]	−1.91	4.20E-04
NT01EI_0487	Anaerobic ribonucleoside-triphosphate reductase, putative	−1.89	6.24E-04
NT01EI_1241	Dyp-type peroxidase family protein	−1.75	7.23E-03
NT01EI_2678	NADH-quinone oxidoreductase subunit B	−1.72	9.52E-03
NT01EI_0391	Fumarate reductase iron-sulfur subunit	−1.71	3.30E-04
NT01EI_0719	3-isopropylmalate dehydrogenase	−1.69	2.49E-03
NT01EI_2672	NADH-quinone oxidoreductase subunit I	−1.67	3.24E-04
NT01EI_1823	Enoyl-[acyl-carrier-protein] reductase [NADH]	−1.65	5.65E-03
NT01EI_0971	Catalase-peroxidase	−1.64	3.84E-03
NT01EI_0659	Bifunctional aspartokinase/homoserine dehydrogenase	−1.56	4.47E-03
NT01EI_1415	Bifunctional polymyxin resistance protein ArnA	−1.51	4.68E-03

In the ¾ MIC TC treatment group, upregulated proteins were enriched in porin activity (7 proteins), flagellar assembly (3 proteins), and ribosomal structure and function (11 proteins) (Table 3). Meanwhile, proteins involved in purine metabolism (9 proteins) were significantly downregulated in *E. ictaluri* exposed to ¾ MIC TC. GO analysis indicated that, after exposure to ¾ MIC of TC, *E. ictaluri* showed enriched upregulation in ribosomal components (11 proteins), non-membrane organelle (16 proteins), outer membrane (11 proteins), and the pore complex (5 proteins), as shown in S4 Table.

**Table 3:** The most significant enriched upregulated and downregulated proteins common in the ¾ MIC-treated *E. ictaluri* sample compared to the non-treated control group.

Locus tag	Description	Fold changes	P.Value
**Upregulation**			
**Porin activity (Go Function, FDR = 0.0241**)			
NT01EI_0220	Maltoporin, putative	8.11	5.50E-06
NT01EI_1875	Gram-negative porin family protein	6.92	5.87E-09
NT01EI_1392	Outer membrane protein A	3.71	1.54E-08
NT01EI_0021	OmpA domain protein	3.29	5.52E-07
NT01EI_3831	Vitamin B12 transporter BtuB	2.08	3.93E-03
NT01EI_1359	Gram-negative porin family protein	1.73	2.11E-04
NT01EI_2856	Peptidoglycan-associated lipoprotein	1.71	9.32E-05
**Flagellar proteins**			
NT01EI_2407	Flagellin	1.34	5.38E-01
flgH	Flagellar L-ring protein	1.17	3.87E-01
NT01EI_2421	Flagellar M-ring protein	1.71	8.18E-01
**Structural constituent of ribosome (KEGG pathway, FDR = 0.0013 and Go Function, FDR = 0.0207)**			
NT01EI_0526	30S ribosomal protein S21	4.11	4.62E-05
NT01EI_3577	30S ribosomal protein S5	3.48	2.65E-04
NT01EI_0593	30S ribosomal protein S9	2.95	3.85E-03
NT01EI_1889	50S ribosomal protein L20	1.84	3.51E-03
NT01EI_3223	30S ribosomal protein S16	1.83	4.08E-03
NT01EI_3588	30S ribosomal protein S3	1.72	5.36E-03
NT01EI_0426	30S ribosomal protein S18	1.72	5.24E-04
NT01EI_3592	50S ribosomal protein L23	1.62	3.26E-03
NT01EI_0170	50S ribosomal protein L11	1.59	4.99E-03
NT01EI_3220	50S ribosomal protein L19	1.55	4.16E-03
**Downregulations**			
**Purine metabolism (KEGG: FDR = 0.0109)**			
NT01EI_1174	Pyrimidine/purine nucleoside phosphorylase	−2.45	5.39E-03
NT01EI_0487	Anaerobic ribonucleoside-triphosphate reductase	−2.39	2.18E-05
NT01EI_0684	Xanthine dehydrogenase, Fe-S subunit, putative	−2.11	6.67E-03
NT01EI_3529	Carbamate kinase	−2.10	1.20E-04
NT01EI_2633	ribonucleoside-diphosphate reductase	−2.03	2.93E-03
NT01EI_0686	Xanthine dehydrogenase, molybdenum-binding subunit	−1.77	3.26E-03
NT01EI_0825	AMP nucleosidase	−1.73	9.64E-05
NT01EI_0912	Xanthine-guanine phosphoribosyltransferase	−1.71	1.74E-03
NT01EI_2893	Phosphoglucomutase, alpha-D-glucose phosphate-specific, putative	−1.61	3.89E-04

### Metabolic remodeling and secretion system repression in TC-adapted *E. ictaluri* strains

To identify the molecular mechanisms underlying microbial adaptation to long-term TC exposure, the proteomes of D30-adapted and D60-adapted *E. ictaluri* strains were compared to a non-adapted control. A total of 124 upregulated and 141 downregulated proteins were commonly found in D30-adapted and D60-adapted strains (Fig 3B). The upregulated proteins were predominantly involved in electron transport and redox metabolism (20 proteins), carbon and energy metabolism (14 proteins), carbohydrate metabolism (7 proteins), nucleotide metabolism (11 proteins), amino acid metabolism (3 proteins), transcription and translation regulation (2 proteins), and transport processes (1 protein) (Table 4). In contrast, most of the downregulated proteins were associated with the Type III and Type VI secretion systems (23 proteins), which are key components of bacterial virulence. Multiple core components and effector proteins of both systems were strongly repressed. This proteomic profile suggests a metabolic shift toward survival and energy conservation, accompanied by a reduction in virulence-associated secretion systems in response to long-term adaptation under TC stress.

**Table 4:** The most significant upregulated and downregulated proteins shared by D30-adapted and D60-adapted strains.

Locus tag	Description	Fold changes D30-adapted	Fold changes D60-adapted
**Upregulation**			
**Electron transport and redox metabolism**			
NT01EI_2476	Dimethylsulfoxide reductase, chain B, putative	10.27	11.39
NT01EI_2477	Anaerobic dimethyl sulfoxide reductase chain A, putative	3.18	3.94
NT01EI_2573	trimethylamine-N-oxide reductase	2.45	2.53
NT01EI_0255	Nitrate reductase, beta subunit, putative	2.11	2.03
NT01EI_0390	Fumarate reductase subunit C	3.76	3.51
NT01EI_0391	Fumarate reductase iron-sulfur subunit	3.61	3.97
NT01EI_0392	Fumarate reductase flavoprotein subunit	2.35	2.87
NT01EI_0686	Xanthine dehydrogenase, molybdenum-binding subunit	3.16	3.27
NT01EI_0684	Xanthine dehydrogenase, Fe-S subunit, putative	2.53	2.23
NT01EI_2670	NADH-quinone oxidoreductase subunit K	1.59	1.88
NT01EI_2679	NADH-quinone oxidoreductase subunit A	1.62	1.80
NT01EI_2572	Cytochrome c-type protein	2.11	2.28
NT01EI_3524	Selenate reductase YgfK, putative	2.08	2.03
NT01EI_3406	Hydrogenase 1 maturation protease, putative	5.98	6.15
NT01EI_3407	Hydrogenase 1 b-type cytochrome subunit, putative	4.06	3.34
NT01EI_3408	Hydrogenase-1 large chain, putative	2.07	1.64
NT01EI_2657	Hydrogenase-2 large chain, putative	2.93	2.69
NT01EI_3283	Hydrogenase accessory protein HypB, putative	2.73	2.25
NT01EI_3178	2Fe-2S ferredoxin	1.58	1.75
NT01EI_3682	Thiosulfate sulfurtransferase GlpE	3.10	2.69
**Central carbon and energy metabolism**			
NT01EI_1605	Pyruvate kinase	1.84	3.41
NT01EI_2386	Fumarate hydratase class I	1.87	1.67
NT01EI_3677	Glycogen synthase	6.11	6.02
NT01EI_3670	Alpha-1,4 glucan phosphorylase	2.66	6.68
NT01EI_3678	Alpha-1,4 glucan phosphorylase	3.39	2.69
NT01EI_3674	1,4-alpha-glucan branching enzyme GlgB	2.55	3.39
NT01EI_3672	Glucose-1-phosphate adenylyltransferase	2.19	2.69
NT01EI_3676	Glucose-1-phosphate adenylyltransferase	1.97	2.08
NT01EI_3748	2-dehydro-3-deoxygluconokinase, putative	1.84	2.27
NT01EI_2842	Galactose-1-phosphate uridylyltransferase	1.67	2.11
NT01EI_3022	Enolase	1.65	1.78
NT01EI_2841	UDP-glucose 4-epimerase	2.19	2.48
NT01EI_2694	Acetate kinase	2.51	2.41
NT01EI_3529	Carbamate kinase	3.07	3.32
**Carbohydrate metabolism**			
NT01EI_0232	Citrate lyase subunit beta	2.69	4.23
NT01EI_3791	Fructose-1,6-bisphosphatase	2.48	2.11
NT01EI_1719	PTS system, mannose/fructose/sorbose family, IID	2.57	2.33
NT01EI_1720	PTS system, mannose/fructose/sorbose family, IIC component	2.45	2.64
NT01EI_1721	PTS system mannose-specific EIIAB component	1.80	2.10
NT01EI_1132	UDP-sugar hydrolase, putative	2.35	2.31
NT01EI_3464	Glycerol-3-phosphate dehydrogenase, anaerobic, C subunit	1.58	1.56
**Nucleotide metabolism**			
NT01EI_0177	Phosphomethylpyrimidine synthase	8.82	6.59
NT01EI_2349	Orotidine 5’-phosphate decarboxylase	2.50	2.31
NT01EI_3478	Aspartate carbamoyltransferase	2.36	1.93
NT01EI_3477	Aspartate carbamoyltransferase regulatory chain	2.45	2.69
NT01EI_0689	Carbamoyl-phosphate synthase small chain	1.57	1.82
NT01EI_0690	Carbamoyl-phosphate synthase large chain	1.60	1.80
NT01EI_0565	Purine nucleoside phosphorylase DeoD-type	1.51	2.01
NT01EI_3530	Dihydropyrimidinase, putative	2.71	2.69
NT01EI_1376	Dihydroorotate dehydrogenase (quinone)	1.73	1.65
NT01EI_2353	GTP cyclohydrolase-2	1.59	1.83
NT01EI_3253	5’/3’-nucleotidase SurE	1.61	1.82
**Ion transport and metal homeostasis**			
NT01EI_2836	Molybdate ABC transporter, periplasmic molybdate-binding protein	3.29	2.00
NT01EI_1879	Ferritin/DPS protein domain-containing protein	2.03	2.22
**Transcription/Translation/RNA Metabolism**			
NT01EI_0790	RNA polymerase-binding transcription factor DksA	1.83	2.23
NT01EI_2560	Ribosomal protein S12 methylthiotransferase RimO	2.30	2.50
NT01EI_0531	Multifunctional CCA protein	2.28	1.92
**Amino acid metabolism**			
NT01EI_0908	Peptidase T, putative	2.58	2.87
NT01EI_0377	Aspartate ammonia-lyase	2.38	2.38
NT01EI_2460	L-asparaginase, type 2, putative	1.52	1.68
**Miscellaneous**			
NT01EI_2443	Amidohydrolase family protein, putative	3.18	3.03
NT01EI_3463	YhcH/YjgK/YiaL family protein	2.99	3.29
NT01EI_0597	YhcH/YjgK/YiaL family protein	3.01	2.97
NT01EI_3531	M20/DapE family protein YgeY, putative	2.81	2.64
**Downregulation**			
**Virulence factors (T3SS and T6SS)**			
NT01EI_2739	Type VI secretion system effector, Hcp1 family	−996	−739.29
NT01EI_0948	Uncharacterized protein	−151.17	−136.24
NT01EI_0950	Type III secretion system effector protein	−125.37	−177.29
NT01EI_0949	Secreted effector protein SseD	−33.13	−19.16
NT01EI_2745	Type VI secretion system Vgr family protein	−43.11	−29.86
NT01EI_0951	Type III secretion low calcium response chaperone LcrH/SycD	−22.16	−34.30
NT01EI_0963	Type III secretion protein, YscU/HrpY family	−21.26	−34.06
NT01EI_2737	Type VI secretion protein, VC_A0107 family	−19.29	−22.47
NT01EI_2749	Type VI secretion protein, VC_A0114 family	−19.16	−10.41
NT01EI_0960	Type III secretion apparatus protein, YscR/HrcR family, putative	−16.91	−13.45
NT01EI_2738	Type VI secretion protein, EvpB/VC_A0108 family	−13.74	−14.42
NT01EI_0943	Type 3 secretion system secretin	−13.36	−12.55
NT01EI_0641	Pentapeptide repeat family protein	−13.18	−20.53
NT01EI_2740	Uncharacterized protein	−12.47	−21.41
NT01EI_2744	Type VI secretion ATPase, ClpV1 family, putative	−11.24	−4.66
NT01EI_0952	EseB secreted effector protein SseB	−11.16	−18.25
NT01EI_0936	Lipoprotein	−9.85	−6.15
NT01EI_0942	Type III secretion apparatus protein, YscD/HrpQ family, putative	−9.38	−10.06
NT01EI_0956	Uncharacterized protein	−9.38	−11.79
NT01EI_0957	Type III secretion ATPase FliI/YscN family, putative	−7.21	−11.16
NT01EI_2747	Type VI secretion-associated protein, ImpA family	−5.70	−7.41
NT01EI_0953	Uncharacterized protein	−4.26	−11.24
NT01EI_2751	IcmF-related N-terminal domain-containing protein	−2.62	−1.74
NT01EI_3574	Protein translocase subunit SecY	−2.75	−2.71

GO enrichment analysis for upregulated proteins in the D30-adapted and D60-adapted *E. ictaluri* strains demonstrated enrichment of energy derivation by oxidation of organic compounds, *de novo* pyrimidine nucleobase biosynthetic process, nucleobase metabolic process, *de novo* pyrimidine and UMP biosynthesis, cellular respiration, metal ion binding, and glycogen biosynthetic process (Table 5). KEGG pathway analysis further highlighted significant upregulation in central metabolic pathways, including pyrimidine metabolism, microbial metabolism in diverse environments, starch and sucrose metabolism, and carbon metabolism (Table 5). STRING cluster analysis strongly reinforced these findings, identifying purine and pyrimidine metabolism as the most significantly affected protein clusters (Table 5).

**Table 5:** Functional enrichments of commonly upregulated proteins in D30-adapted and D60-adapted strains.

Category	Term description	Group	Count	FDR
**Upregulated proteins in D30-adapted and D60-adapted strains**				
GO Process	Generation of precursor metabolites and energy	D-30	23	7.70E-04
		D-60	29	2.20E-04
	Energy derivation by oxidation of organic compounds	D-30	18	7.70E-04
		D-60	19	3.80E-03
	De novo pyrimidine nucleobase biosynthetic process	D-30	5	1.57E-02
		D-60	5	2.21E-02
	Nucleobase metabolic process	D-30	10	7.70E-04
		D-60	9	1.31E-02
	De novo UMP biosynthetic process	D-30	5	1.57E-02
		D-60	6	1.31E-02
	Cellular respiration	D-30	14	1.57E-02
		D-60	15	2.21E-02
	UMP metabolic process	D-30	6	1.57E-02
		D-60	7	1.31E-02
	Glycogen biosynthetic process	D-30	4	1.61E-02
		D-60	4	3.63E-02
GO Function	Metal ion binding	D-30	49	1.70E-04
		D-60	52	4.36E-02
KEGG pathway	Metabolic pathways	D-30	58	9.45E-05
		D-60	73	1.49E-05
	Pyrimidine metabolism	D-30	11	3.00E-04
		D-60	11	3.60E-03
	Starch and sucrose metabolism	D-30	6	1.53E-02
		D-60	6	3.10E-02
	Microbial metabolism in diverse environments	D-30	24	3.00E-04
		D-60	25	4.80E-03
	Alanine, aspartate, and glutamate metabolism	D-30	7	1.81E-02
		D-60	7	4.60E-02
	Carbon metabolism	D-30	12	3.17E-02
		D-60	15	1.64E-02
String Cluster	Purine and pyrimidine metabolism	D-30	23	2.43E-06
		D-60	24	7.16E-05
**Downregulated proteins in D30-adapted and D60-adapted strains**				
GO Process	Protein transmembrane transport	D-30	13	7.30E-04
		D-60	18	8.15E-06
	Protein transport	D-30	16	1.29E-02
		D-60	23	2.23E-05
STRING clusters	Protein secretion and tetratricopeptide TPR-3	D-30	11	3.65E-05
		D-60	11	2.00E-04
	Protein transmembrane transport, and Hcp1-like superfamily	D-30	9	1.50E-03
		D-60	11	2.50E-04
	Biosynthesis of amino acids, and Glutathione metabolism	D-30	17	3.43E-02
		D-60	20	2.51E-02
KEGG	Bacterial secretion system	D-30	10	2.02E-02
		D-60	14	2.80E-04

GO enrichment analysis for downregulated proteins revealed that protein secretion and protein transmembrane transport were enriched in D30-adapted and D60-adapted strains. KEGG analysis further showed that the bacterial secretion system pathway, specifically (T3SS and T6SS), was significantly downregulated in both adapted strains compared to the control group (Table 5).

### Specific proteomic changes for D30- and D60-adapted strains

In the D30-adapted strain, enrichment analysis revealed an upregulation of proteins related to methane metabolism (6 proteins), biosynthesis of secondary metabolites (26 proteins), nitrotoluene degradation (3 proteins), purine metabolism (9 proteins), and pyruvate metabolism (7 proteins) (S5 Table). These findings suggest that the bacteria are primarily focused on processes related to their own metabolism and survival, rather than a specific mechanism of action against the drug.

In the D60-adapted strain, seven proteins related to the citrate cycle (TCA cycle) pathway and 10 proteins involved in the amino sugar and nucleotide metabolism pathway were significantly upregulated compared to the control group (Table 6). The upregulated TCA cycle proteins include subunits of fumarate reductase (iron-sulfur, flavoprotein, and subunit C), as well as fumarate hydratase (class I and II), a pyruvate dehydrogenase complex component, and succinate dehydrogenase iron-sulfur subunit. Proteins in the amino sugar and nucleotide sugar metabolism pathway included multiple components of the mannose/fructose-specific PTS transport system, enzymes involved in UDP-glucose and galactose metabolism (e.g., UDP-glucose 4-epimerase and galactose-1-phosphate uridylyltransferase), and key enzymes in glycogen biosynthesis. This strong upregulation suggests that the D-60 strain enhances carbohydrate uptake, processing, and energy generation as part of its adaptive response to TC stress.

**Table 6:** Enriched upregulated KEGG pathways and GO functions in the D60-adapted strain.

Locus tag	Description	Fold changes	P.Value
**Citrate cycle (TCA cycle), FDR of= 0.0293**			
NT01EI_0391	Fumarate reductase iron-sulfur subunit	3.97	1.27E-09
NT01EI_0390	Fumarate reductase subunit C	3.51	4.03E-05
NT01EI_0392	Fumarate reductase flavoprotein subunit	2.87	3.19E-08
NT01EI_2386	Fumarate hydratase class I	1.67	9.23E-05
NT01EI_2078	Fumarate hydratase class II	1.64	1.69E-03
NT01EI_0759	Acetyltransferase component of pyruvate dehydrogenase complex	1.52	8.59E-04
NT01EI_2869	Succinate dehydrogenase iron-sulfur subunit	1.51	7.53E-03
**Amino sugar and nucleotide sugar metabolism pathway, FDR = 0.0152**			
NT01EI_1719	PTS system, mannose/fructose/sorbose family, IID component	2.33	1.11E-06
NT01EI_1720	PTS system, mannose/fructose/sorbose family, IIC component	2.64	1.04E-04
NT01EI_1721	PTS system mannose-specific EIIAB component	2.10	5.06E-06
NT01EI_2841	UDP-glucose 4-epimerase	2.48	2.21E-06
NT01EI_2842	Galactose-1-phosphate uridylyltransferase	2.11	1.03E-03
NT01EI_3672	Glucose-1-phosphate adenylyltransferase	2.69	2.02E-03
NT01EI_3676	Glucose-1-phosphate adenylyltransferase	2.08	3.48E-08
NT01EI_3915	Glutamine--fructose-6-phosphate aminotransferase [isomerizing]	1.68	2.63E-05
NT01EI_0995	N-acetylmuramic acid 6-phosphate etherase	1.60	1.69E-02
NT01EI_1311	UDP-glucose 4-epimerase	3.18	6.06E-03

GO analysis of downregulated proteins in the D60-adapted strain showed reduced expression in functional categories, including ATP-dependent activity, nucleotide and small molecule binding, ATP hydrolysis, purine ribonucleotide binding, purine ribonucleoside triphosphate binding, anion binding, and carbohydrate derivative binding (S6 Table), suggesting a shift in energy utilization and regulatory processes.

### Between-group enrichment analysis

**Fig 4:**
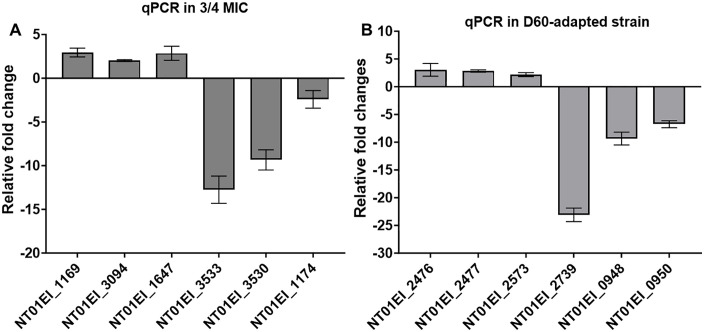
qRT-PCR validation of proteomic responses. **(A)** Relative transcript levels in wild-type exposed to ¾ MIC TC (90 µg/mL) relative to unexposed control. **(B)** Relative transcript levels in the D60-TC–adapted strain relative to the adapted control. Bars show mean fold change ± SEM from n = 3 independent cultures. Expression was normalized to 16S rRNA. Representative genes were selected from proteomic hits that represent key pathways, including efflux pumps, nucleotide biosynthesis, electron transport, and virulence factors.

Enrichment analysis revealed that ten proteins were consistently downregulated in all TC-exposed groups, including ^2^/_4_ MIC, ¾ MIC, D30-adapted, and D60-adapted strains, when compared to the control group. These proteins were primarily associated with riboflavin biosynthesis (two key enzymes), outer membrane protein integrity (enterobacterial TraT), stress adaptation (universal stress protein), oxidative stress defense (superoxide dismutase), and DNA repair (DNA ligase) (S7 Table). In addition, eight proteins related to purine and pyrimidine metabolism were downregulated in the ^2^/_4_ MIC and ¾ MIC groups but upregulated in the D30-adapted and D60-adapted strains, as identified through enrichment analysis and STRING (S7 Table). This pattern suggests that purine and pyrimidine metabolism is influenced by both time and concentration levels of TC exposure. To validate the proteomic findings, we performed qRT-PCR on transcripts corresponding to a subset of differentially abundant proteins. The qRT-PCR results confirmed the proteomic trends, confirming the observed expression patterns (Fig 4).

### Shared and strain-specific mutations in TC-adapted *E. ictaluri* strains

We assembled the genomes of the *E. ictaluri* D30-TC, D60-TC, D30-BHI, D60-BHI, and parental strains to investigate genomic changes associated with TC adaptation. Comparative analysis revealed multiple mutations across the adapted strains, including SNPs, insertions, deletions, and complex variations affecting a broad range of functional genes. Several genes carried mutations in both the D30-TC and D60-TC strains when compared to their respective controls (D30-BHI and D60-BHI), indicating common adaptive responses to TC exposure (Table 7). Among these were genes involved in membrane biosynthesis (apolipoprotein N-acyltransferase and F0F1 ATP synthase), energy metabolism (F0F1 ATP synthase), metabolic enzymes (bifunctional glucose-1-phosphatase/inositol phosphatase, glycogen debranching protein GlgX, and maltodextrin phosphorylase), and nucleotide and cell division regulation genes (partition protein MukF and LPD38 domain-containing protein). Additionally, genes involved in stress response and environmental sensing (ribose operon repressor RbsR and two-component system response regulator PhoP), and anaerobic respiration and redox metabolism (TMAO reductase system protein TorT) were commonly affected in both strains. The recurrence of mutations in these functional categories across both adapted strains underscores their likely importance in facilitating survival and physiological adjustment under prolonged TC exposure.

**Table 7:** Gene products shared between D30-TC and D60-TC strains compared to their respective controls (D30-BHI and D60-BHI).

Gene Product	Shared in both strains	Type of mutation
Apolipoprotein N-acyltransferase	Yes	SNP (T → C/ G → A)
Bifunctional glucose-1-phosphatase/inositol phosphatase	Yes	del
Bifunctional hydroxymethylpyrimidine kinase/phosphomethylpyrimidine kinase	Yes	SNP (G → T/ C → A)
Chromosome partition protein MukF	Yes	SNP (A → G/ T → C)
F0F1 ATP synthase subunit A	Yes	SNP (C → T/ A → G)
Glycine dehydrogenase (decarboxylating)	Yes	SNP (G → A/ T → C)
Glycogen debranching protein GlgX	Yes	SNP (T → C/ G → A)
LPD38 domain-containing protein	Yes	SNP (A → G/ C → T)
Maltodextrin phosphorylase	Yes	(del)
N-acetylmuramoyl-L-alanine amidase AmiC	Yes	SNP (T → G/ A → C)
Phosphate transport regulator	Yes	del)
Ribose operon transcriptional repressor RbsR	Yes	SNP (T → C/ A → G)
Sulfatase	Yes	SNP (C → T/ A → G)
TMAO reductase system periplasmic protein TorT	Yes	del/ins
Two-component system response regulator PhoP	Yes	SNP (G → A/ C → T)

In the D30-TC strain, a total of 69 mutations were identified, comprising primarily SNPs, insertions, deletions, and complex mutations (S8 Table). These genetic alterations were distributed among genes essential for a variety of cellular functions, such as central carbohydrate and energy metabolism (20 genes), stress response and signaling genes (6 genes), ribosomal function (6 genes), amino acid metabolism (3 genes), cell wall biosynthesis and membrane proteins (10 genes), regulatory and transcription (6 genes), and DNA replication and repair (10 genes).

In the D60-TC strain, unique alterations were found in genes involved in cellular processes associated with resistance, survival, and metabolic efficiency under antibiotic stress (S9 Table). Mutations were found in genes involved in cell envelope biosynthesis, nutrient transport, and protein translocation across membranes (11 genes), metabolic activities and energy production (15 genes), transcriptional regulators that control expression of stress response (5 genes), genome stability and chromosome segregation (7 genes), antimicrobial resistance and stress response (5 genes), ribosomal function (1 gene), and hypothetical/uncharacterized (5 genes). Together, these exclusive mutations in D30-TC and D60-TC reflect extended adaptation strategies involving metabolic shifts, maintenance of membrane integrity, stress signaling, and remodeling of the regulatory network in response to sustained TC exposure.

## Discussion

Essential oils and their active components are gaining attention as alternative strategies to combat bacterial infections, particularly in the face of rising antibiotic resistance. Trans-cinnamaldehyde (TC) has shown promising efficacy against a range of bacterial pathogens, including *E. ictaluri*. However, the adaptive responses of bacteria to prolonged TC exposure remain poorly understood and may compromise treatment efficacy. In this study, we investigated the mechanisms of TC action and examined the molecular consequences of prolonged TC exposure in *E. ictaluri* to uncover mechanisms of tolerance and identify potential implications for future therapeutic interventions.

Although the MIC values for TC remained unchanged in the D30-TC and D60-TC adapted strains, inhibition zone assays revealed notable shifts in antimicrobial susceptibility, highlighting the impact of prolonged TC exposure on the antibiotic response profile of *E. ictaluri*. Both D30-TC and D60-TC strains exhibited reduced susceptibility to TC and florfenicol, as evidenced by smaller inhibition zones compared to the wild-type and BHI-adapted controls. This reduction may reflect adaptive physiological or genomic modifications, such as changes in membrane composition or permeability, alterations in transmembrane transport systems, mutations in antibiotic target sites, or activation of efflux pumps, which could confer a survival advantage under antibiotic pressure. Conversely, both D30-TC and D60-TC strains display enhanced susceptibility to SXT, suggesting a potential trade-off in adaptive tolerance mechanisms, possibly due to shifts in metabolic pathways or regulatory networks affected during adaptation to TC. It is plausible that changes acquired to manage TC stress inadvertently increased sensitivity to SXT, reflecting complex cross-tolerance or collateral sensitivity relationships. These findings underscore the complexity of bacterial adaptation and emphasize the need to consider such dynamics when developing treatment strategies and managing antibiotic stewardship in aquaculture settings.

Research on the impact of TC on bacterial efflux systems demonstrates a complex and variable response. In *Pseudomonas aeruginosa* PA14, sub-inhibitory concentrations of cinnamaldehyde induced a strong transient upregulation of RND family efflux pumps, leading to increased resistance to antibiotics like meropenem and ciprofloxacin [38]. Conversely, in methicillin-resistant *Staphylococcus aureus* (MRSA), TC significantly increased susceptibility to gentamicin by downregulating resistance genes (*mecA, mecR1,* and *mecI*) and biofilm-associated regulators [39]. Together, these results suggest that while long-term TC adaptation may promote resistance to certain antibiotics, it may also inadvertently create vulnerabilities to others. In the present study, exposure of the *E. ictaluri* 93–146 strain to two different levels of TC resulted in the upregulation of efflux-related proteins and outer membrane proteins. This observation aligns with prior evidence that efflux systems are critical components of bacterial adaptive responses to environmental stressors [40,41]. Efflux pump expression is commonly induced by oxidative stress, membrane damage, and nitrosative or disulfide stress. While the transcriptional activation of efflux genes under such challenging conditions is well documented, the extent to which this heightened efflux activity contributes to antibiotic tolerance remains poorly understood. The upregulation of these proteins under TC exposure suggests an integrated response that includes enhanced drug export and detoxification capacity. Previous studies on *P. aeruginosa* have shown enhanced efflux activity upon exposure to high concentrations of cinnamaldehyde, particularly through activation of the MexAB-OprM RND-type efflux system [26], resulting in an increased expulsion of drugs and a subsequent decrease in the effectiveness of antibiotics. This stimulation was found to be transient, persisting only until cinnamaldehyde degraded. Moreover, sustained resistance in *P. aeruginosa* was only observed after several days of exposure to concentrations exceeding 900 µg/mL, above the reported MIC of 700 µg/mL [42,43]. Another study on *Acinetobacter baumannii* exposed to sub-MIC of TC reported significant downregulation of the efflux pump genes *adeA* and *adeB* [44]. This context dependence provides a mechanistic rationale for our observation that TC-adapted *E. ictaluri* showed decreased zones for florfenicol yet increased sensitivity to SXT, a pattern of collateral sensitivity arising from network-level stress responses rather than fixed target mutations.

Interestingly, numerous proteins involved in purine and pyrimidine metabolism were consistently downregulated in both ^2^/_4_ and ^3^/_4_ MIC-treated groups, indicating a disruption in nucleotide metabolism. Such alteration can have far-reaching physiological consequences, including impaired DNA synthesis and repair, accumulation of DNA damage, and overproduction of reactive oxygen species (ROS), which collectively contribute to oxidative stress and cellular damage [45]. These findings suggest that sub-MIC concentrations of TC can compromise the metabolic fitness of *E. ictaluri*, potentially limiting its ability to replicate, maintain genomic integrity, and survive under stress conditions. Given that purine and pyrimidine biosynthesis are highly energy-intensive processes, their suppression may reflect a deliberate adaptation to conserve resources under TC stress. By downregulating these pathways, the pathogen may reduce its overall energy expenditure, prioritizing defense mechanisms overgrowth and replication. The coordinated downregulation of DNA repair and antioxidant proteins likely explains the observed loss of virulence in D30 and D60-adapted strains, as impaired DNA repair compromises the bacteria’s ability to maintain genome integrity in the face of host-induced oxidative stress, and weakened antioxidant defenses leave the bacteria vulnerable to reactive oxygen species produced by the host immune system.

Exposure of *E. ictaluri* to ^2^/_4_ and ^3^/_4_ MIC concentrations of TC resulted in the downregulation of proteins essential for genome maintenance, redox homeostasis, and metabolism. Notably, proteins involved in DNA repair and protein quality control (Clp proteases and ClpB) were reduced. This suggests that TC may compromise the ability of *E. ictaluri* to repair damage and maintain functional proteins. In parallel, multiple redox-active enzymes showed marked downregulation, indicating that the *E. ictaluri* ability to manage oxidative stress and maintain redox balance may be impaired, potential leading to an increase in oxidative stress. Additionally, downregulation of enzymes involved in amino acid and nitrogen metabolism, metal ion storage, and energy production reflects a general suppression of metabolic and biosynthetic activity, likely as a stress adaptation to minimize energy expenditure. Reductions in riboflavin biosynthesis, carbohydrate metabolism, and membrane-associated stress response proteins further underscore a global metabolic shift. Collectively, these findings suggest that TC compromises the *E. ictaluri* ability to repair damage, maintain protein quality, manage oxidative stress, and maintain metabolic functions, with potential consequences on growth and stress tolerance. This supports the conclusion that the antimicrobial action of TC involves interference with essential cellular processes critical for bacterial survival and adaptation. Previous proteomic analyses support these findings, showing TC disrupts multiple essential pathways, including disruption of amino acid, carbohydrate, and lipid metabolism, resulting in the inhibition of cellular defenses against oxidative stress [46]. This is further supported by studies on aquaculture pathogens such as *Aeromonas hydrophila*, where cinnamaldehyde increases membrane permeability and damages cell morphology, leading to leakage of cellular contents [47]. In another study, cinnamaldehyde was found to downregulate quorum sensing-related genes affecting virulence factors like aerolysin and biofilm formation, reducing pathogenicity [48].

Proteomic analysis revealed that ¾ MIC of TC resulted in the upregulation of several proteins involved in both ribosome biogenesis and flagellar assembly, indicating enhanced protein synthesis and potential changes in motility at higher TC concentrations. Interestingly, these changes were absent in the ^2^/_4_ MIC group, suggesting that the impact of TC on *E. ictaluri* is dose-dependent, with higher sub-MIC levels triggering compensatory responses that may support protein production and motility, potentially as a stress adaptation mechanism to maintain cellular function or facilitate escape from hostile conditions.

Adaptation of *E. ictaluri* to TC over 30 and 60 days resulted in a significant upregulation of proteins involved in electron transport and redox metabolism, indicating a metabolic shift toward enhanced anaerobic respiration and redox homeostasis, likely as an adaptive response to prolonged TC stress. This shift suggests that prolonged exposure to low levels of TC disrupts aerobic energy metabolism, prompting the bacterium to activate alternative energy pathways to maintain ATP production under stress conditions. Additionally, the increased abundance of hydrogenase complexes and accessory proteins points to a shift toward hydrogen metabolism, which can support redox balancing and energy generation when conventional respiration is compromised. With the unchanged MIC and preserved growth kinetics, these proteomic patterns suggest that the loss of virulence results from the regulatory reprogramming of energy and redox networks, rather than from the direct bactericidal activity of TC. To our knowledge, this is a novel mechanism by which a phytochemical attenuates bacterial virulence. Collectively, these proteomic changes highlight coordinated metabolic modifications, enabling *E. ictaluri* to sustain energy production and redox balance in response to TC-induced membrane perturbation and oxidative stress.

Another important finding in this study was pronounced upregulation of proteins involved in central carbon and energy metabolism in both D30 and D60 TC-adapted *E. ictaluri* strains, indicating a strategic metabolic reprogramming to support long-term survival under TC stress. Notably, enhanced glycogen and glycolytic flux enzyme expression in both D30 and D60 adapted strains suggests a strategy for rapid ATP generation under nutrient-limited or stress conditions. This metabolic shift likely supports increased energy production and provides essential intermediates for cellular adaptation and repair. Additionally, the upregulation of nucleotide sugar metabolism enzymes suggests an increase in the synthesis of cell wall precursors, which may help reinforce membrane integrity. Importantly, increases in proteins related to transcription, translation, amino acid metabolism, and multiple components of the phosphotransferase system (PTS) suggest a broad metabolic adaptation to fluctuating nutrient availability under TC stress. The upregulation of ion transport and metal homeostasis proteins suggests an adaptive response to maintain essential metal ion pools and protect against oxidative damage, which is critical for enzymatic function and cellular integrity during antimicrobial challenge. Together, these changes illustrate a flexible and robust metabolic response that enables *E. ictaluri* to persist and adapt in the presence of TC, while also highlighting potential vulnerabilities, such as its reliance on glycogen and fermentation pathways, that could be exploited to enhance antimicrobial strategies.

Long-term exposure of *E. ictaluri* to TC led to a marked reduction in virulence, as both the D30- and D60-adapted strains caused no mortality in catfish, unlike the wild-type strain, which induced over 80% mortality. This finding aligns with proteomic data, which show significant downregulation of key virulence-related proteins, particularly the T6SS and T3SS systems. These systems are critical virulence mechanisms in *E. ictaluri*, enabling the bacterium to inject effector proteins directly into host cells, disrupt host immune responses, and promote bacterial survival and replication within the host [49–51]. The downregulation of these systems is likely to be a major contributing factor to the decreased pathogenicity of D30 and D60-adapted strains by impairing the injection of effector proteins into host cells. Importantly, since a low TC concentration was used in this experiment, the reduction in virulence is not attributable to bactericidal activity, but rather to modulation of gene expression and regulatory networks. Similarly, a previous study reported that cinnamaldehyde reduced virulence in *Listeria monocytogenes* by downregulating the transcription of the virulence factors [52], and suppressed T3SS/T6SS and quorum sensing in *Acinetobacter baumannii* and *Clostridioides difficile* [44,53]. Together, these results suggest that prolonged exposure to low doses of TC can attenuate bacterial virulence without killing the pathogen, offering insights into alternative strategies for controlling bacterial infections.

The upregulation of purine and pyrimidine metabolism observed in D30- and D60-adapted strains may represent a compensatory mechanism by which *E. ictaluri* attempts to repair damage caused by TC exposure. TC has been shown to cause DNA damage in bacteria [54], and upregulation of purine and pyrimidine metabolism could enhance the synthesis of nucleotides required for DNA repair and replication. The fact that 8 proteins involved in purine and pyrimidine metabolism are common between all groups suggests that these proteins may play a key role in the adaptive response to TC exposure. Further research is needed to determine the specific roles of these proteins in TC resistance.

By sequencing the genomes of D30-TC and D60-TC strains, along with their respective BHI controls and parental strains, we identified a broad spectrum of mutations. Common mutations were found in genes associated with membrane biosynthesis and modification, including apolipoprotein N-acyltransferase and F0F1 ATP synthase. These proteins are critical for maintaining membrane integrity and cellular energy production, which are essential for bacterial adaptation to membrane-targeting antimicrobial compounds [55,56]. Mutations in metabolic enzymes such as bifunctional glucose-1-phosphatase/inositol phosphatase, glycogen debranching protein GlgX, and maltodextrin phosphorylase suggest a shift in carbohydrate metabolism to optimize energy utilization under stress. Regulatory proteins involved in nucleotide metabolism, cell division, and environmental sensing, including MukF, LPD38, RbsR, and two-component system regulator, also carried mutations in both adapted strains. Such regulators are known to orchestrate bacterial stress responses and facilitate rapid adaptation to changing environments [57,58]. Additionally, mutations affecting anaerobic respiration components, such as the TMAO reductase system protein TorT, suggest a metabolic shift to anaerobic respiration and redox regulation, likely supporting survival under TC-induced oxidative stress, a response similarly observed in other bacteria under antimicrobial pressure [59].

This study has few limitations that guide directions of future work. For example, we only evaluated a single *E. ictaluri* 93–146 strain, so including a broader range of strains would help increase confidence in the findings. Also, an adaptation experiment was performed at one sub-MIC, testing additional concentrations could clarify how responses vary with dose. Our method of daily serial passaging in defined broth is a standard laboratory model, but it does not fully mimic the complex, changing conditions of actual pond environments, which include factors such as environmental stress and interactions with other microbes. Future studies using field-mimicking setups or on-farm trials could provide more realistic insights. Lastly, we assessed virulence and protection at only one immersion challenge dose. Addressing these points in future research will strengthen the practical relevance and robustness of conclusions regarding TC resistance and management.

In conclusion, this study reveals that exposure of *E. ictaluri* to TC at ^2^/_4_ MIC and ^3^/_4_ MIC downregulated critical cellular processes, including DNA repair, protein quality control, redox homeostasis, and core metabolic pathways. These impairments likely lead to the accumulation of reactive oxygen species (ROS), loss of membrane potential, energy depletion, and cellular damage, ultimately inhibiting growth and viability. While long-term TC exposure did not alter the MIC, it triggered significant shifts in the proteome and genome, including upregulation of central metabolic enzymes involved in glycolysis, glycogen metabolism, and redox balance. However, this adaptation came at the cost of reduced virulence, as evidenced by downregulation of key virulence factors (T6SS and T3SS components) and loss of pathogenicity. Notably, the loss of virulence and potential immunogenicity of the TC-adapted strains emphasizes their potential as live-attenuated vaccine candidates. However, this translational implication remains provisional; more rigorous testing is necessary to verify safety, stability, appropriate dosage, and effectiveness across various field settings before confident application. Whole-genome sequencing further revealed shared and unique mutations in key functional genes associated with membrane biosynthesis, metabolism, and regulatory networks, suggesting a multifaceted adaptation strategy. Together, these findings not only advance our understanding of TC’s bacteriostatic and potentially bactericidal mechanisms but also inform its potential use in sustainable aquaculture and antimicrobial stewardship.

## Supporting information

S1 Table Primer sequences used for qRT-PCR validation of proteomic responses. The table lists gene targets selected from key functional pathways, along with their corresponding forward and reverse primer sequences. (PDF)

S2 Table Unique and shared differentially expressed proteins (DEPs) proteins under TC exposure and adaptation. Counts mirror the Venn diagram sectors (Fig 3A and B).
(PDF)

S3 Table Enriched clusters among common downregulated and upregulated proteins in ^2^/_4_ MIC and ^3^/_4_ MIC groups compared to control. (PDF)

S4 Table Most significant enriched upregulated GO components in ¾ MIC sample. (PDF)

S5 Table Enriched upregulated KGEE pathways in D30-TC adapted strain. (PDF)

S6 Table Enriched downregulated GO functions in D60-TC adapted strain. (PDF)

S7 Table Major proteins differentially expressed in *E. ictaluri* strains exposed to ^2^/_4_ MIC, ¾ MIC, D30-adapted, and D60-adapted strains compared to the control group. Notably, proteins involved in purine and pyrimidine metabolism were consistently downregulated in sub-MIC conditions (^2^/_4_ MIC and ^3^/_4_ MIC), while they were upregulated in the D30- and D60-adapted strains. (PDF)

S8 Table Identified SNPs and indels in the D30-TC strain compared to the D30-BHI control. (PDF)

S9 Table Identified alteration in the D60-TC strain compared to the D60-BHI control. (PDF)
