# Supplementary material for: Investigation mechanisms of action and resistance of Edwardsiella ictaluri to trans-cinnamaldehyde
Source: PLoS One. 2026 Jan 7;21(1):e0340053. doi: 10.1371/journal.pone.0340053 (PMC12779148; doi:10.1371/journal.pone.0340053)
Supplement: S1 Table — (PDF) [file pone.0340053.s001.pdf]

S1 Table. Primer sequences used for qRT-PCR validation of proteomic responses. The table lists gene targets selected from key functional pathways, along with their corresponding forward and reverse primer sequences**.**

Locus tag	Forward	Reverse
NT01EI_1169	CAGGACAAAGGGACAGAGATG	GATTTGGCGTTGACCAAAGG
NT01EI_3094	ATCAGGCGCTAATCCTGAATAC	TACACTACGCTGGGAGACATA
NT01EI_1647	CGCTGATCGTTACGGTGATT	CTGCGCCAGATACCGTTAAA
NT01EI_3533	GTAGAGGTCGCCCACAATAA	GCCCAGGACTTGGGATAAA
NT01EI_3530	TGGCGGTACAACAACCATTA	CGTAGTCGATCACTGCCTTATC
NT01EI_1174	AGGGCGAGTATACCTTTACCA	GGAGTAAACACCTGCCAGTC
NT01EI_2476	GAGCTGGCCTGTAAGGATTA	TCGAGAGATAGTAGGCAAACAC
NT01EI_2477	CTACGGTAACGAGGCCATTT	GTCGCCATAGTGGTTGAGATAA
NT01EI_2573	GGAGACTATTCGCAGCTGTATG	CTTCATCCTTACCCTCGCTAAAG
NT01EI_2739	AGGCCACAATGTCGGTATTC	CCTGGGTACAAATCTCCACTTC
NT01EI_0948	GGCCGTGATCGCTCTTATG	GGCGTCGTCGCATCTTATC
NT01EI_0950	GTCATGGCGGGTAAGTCTATC	CAACCTCCTTGCCTATCTCTTT
16s rRNA	AGAAGAAGCACCGGCTAACT	GGATGCAGTTCCCAGGTTAA
