# Supplementary material for: Investigation mechanisms of action and resistance of Edwardsiella ictaluri to trans-cinnamaldehyde
Source: PLoS One. 2026 Jan 7;21(1):e0340053. doi: 10.1371/journal.pone.0340053 (PMC12779148; doi:10.1371/journal.pone.0340053)
Supplement: S2 Table — Counts mirror the Venn diagram sectors (Fig 3A and B). (PDF) [file pone.0340053.s002.pdf]

S2 Table. Unique and shared differentially expressed proteins (DEPs) proteins under TC exposure and adaptation. Counts mirror the Venn diagram sectors (Fig. 3A and B).

**Category**	**3/4 MIC up**	**2/4 MIC up**	**Shared up (both)**	**3/4 MIC down**	**2/4 MIC down**	**Shared Down (both)**	**Mixed direction***
Count	57	59	67	38	77	60	1
**Category**	**D-60 up**	**D-30 up**	**Shared up (both)**	**D-60 down**	**D-30 down**	**Shared down (both)**	**Mixed direction***
Count	66	25	124	80	44	141	0

*Proteins significant but with opposite direction.
