# Supplementary material for: Investigation mechanisms of action and resistance of Edwardsiella ictaluri to trans-cinnamaldehyde
Source: PLoS One. 2026 Jan 7;21(1):e0340053. doi: 10.1371/journal.pone.0340053 (PMC12779148; doi:10.1371/journal.pone.0340053)
Supplement: S3 Table — (PDF) [file pone.0340053.s003.pdf]

S3 Table. Enriched clusters among common downregulated and upregulated proteins in $^{2}/_{4}$ MIC and $^{3}/_{4}$ MIC groups compared to control.

Category	Term Description	Group	FDR	Count
**Upregulated proteins in $^{2}/_{4}$ MIC and $^{3}/_{4}$ MIC group**				
STRING clusters	RND efflux pump, membrane fusion protein, barrel-sandwich domain, and efflux transmembrane transporter	$^{3}/_{4}$ MIC	2.0E-02	8
		$^{2}/_{4}$ MIC	1.6E-02	8
**Downregulated proteins in $^{2}/_{4}$ MIC and $^{3}/_{4}$ MIC group**				
STRING clusters	Purine nucleobase metabolic process and amidohydrolase	$^{3}/_{4}$ MIC	4.6E-04	7
		$^{2}/_{4}$ MIC	1.6E-05	10
STRING clusters	Purine and pyrimidine metabolism	$^{3}/_{4}$ MIC	7.8E-04	14
		$^{2}/_{4}$ MIC	1.1E-04	19
Go Function	Catalytic activity	$^{3}/_{4}$ MIC	2.6E-02	67
		$^{2}/_{4}$ MIC	3.8E-02	89
