# Supplementary material for: Investigation mechanisms of action and resistance of Edwardsiella ictaluri to trans-cinnamaldehyde
Source: PLoS One. 2026 Jan 7;21(1):e0340053. doi: 10.1371/journal.pone.0340053 (PMC12779148; doi:10.1371/journal.pone.0340053)
Supplement: S4 Table — (PDF) [file pone.0340053.s004.pdf]

S4 Table. Most significant enriched upregulated GO components in ¾ MIC sample.

GO component	Count	FDR
Ribosome	11	5.9E-03
Non-membrane-bounded organelle	16	5.9E-03
Intracellular non-membrane-bounded organelle	13	5.9E-03
Pore complex	5	5.9E-03
Outer membrane	11	9.3E-03
