# Supplementary material for: Investigation mechanisms of action and resistance of Edwardsiella ictaluri to trans-cinnamaldehyde
Source: PLoS One. 2026 Jan 7;21(1):e0340053. doi: 10.1371/journal.pone.0340053 (PMC12779148; doi:10.1371/journal.pone.0340053)
Supplement: S5 Table — (PDF) [file pone.0340053.s005.pdf]

S5 Table. Enriched upregulated KGEE pathways in D30-TC adapted strain.

**Kegg Pathway**	**Count**	**FDR**
Methane metabolism	6	3.2E-02
Biosynthesis of secondary metabolites	26	3.2E-02
Nitrotoluene degradation	3	3.4E-02
Purine metabolism	9	4.5E-02
Pyruvate metabolism	7	4.9E-02
