# Supplementary material for: Investigation mechanisms of action and resistance of Edwardsiella ictaluri to trans-cinnamaldehyde
Source: PLoS One. 2026 Jan 7;21(1):e0340053. doi: 10.1371/journal.pone.0340053 (PMC12779148; doi:10.1371/journal.pone.0340053)
Supplement: S6 Table — (PDF) [file pone.0340053.s006.pdf]

S6 Table. Enriched downregulated GO functions in D60-TC adapted strain.

Go Function	Count	FDR
ATP-dependent activity	26	8.2E-04
Small molecule binding	62	9.0E-03
Nucleotide binding	54	3.6E-02
ATP binding	41	3.9E-02
ATP hydrolysis activity	10	3.9E-02
Ribonucleotide binding	44	3.9E-02
Purine ribonucleotide binding	43	3.9E-02
Purine ribonucleoside triphosphate binding	43	3.9E-02
Anion binding	52	3.9E-02
Carbohydrate derivative binding	46	3.9E-02
