# Supplementary material for: Investigation mechanisms of action and resistance of Edwardsiella ictaluri to trans-cinnamaldehyde
Source: PLoS One. 2026 Jan 7;21(1):e0340053. doi: 10.1371/journal.pone.0340053 (PMC12779148; doi:10.1371/journal.pone.0340053)
Supplement: S7 Table — (PDF) [file pone.0340053.s007.pdf]

S7 Table. Major proteins differentially expressed in *E. ictaluri* strains exposed to $^{2}/4$ MIC, ¾ MIC, D30-adapted, and D60-adapted strains compared to the control group. Notably, proteins involved in purine and pyrimidine metabolism were consistently downregulated in sub-MIC conditions ($^{2}/4$ MIC and $^{3}/4$ MIC), while they were upregulated in the D30- and D60-adapted strains.

**Locus tag**	**Description**	**Fold changes**			
		**¾ MIC**	**$^{2}/4$ MIC**	**D-30**	**D-60**
NT01EI_2312	Uncharacterized protein	-4.44	-2.10	-4.06	-298.17
NT01EI_1057	6,7-dimethyl-8-ribityllumazine synthase	-2.75	-2.16	-3.68	-3.20
NT01EI_1751	Universal stress protein	-2.60	-3.20	-2.00	-1.71
NT01EI_0252	Superoxide dismutase [Cu-Zn]	-2.51	-1.91	-2.23	-2.81
NT01EI_2170	3-isopropylmalate dehydratase large subunit	-1.97	-1.64	-4.56	-4.03
NT01EI_1056	Riboflavin biosynthesis protein RibD	-1.96	-2.03	-3.16	-4.86
NT01EI_1249	DNA ligase	-1.84	-1.79	-2.08	-1.87
NT01EI_3205	Chaperone protein ClpB	-1.83	-1.71	-1.75	-2.16
NT01EI_0982	Enterobacterial TraT complement resistance	-1.71	-2.77	-1.69	-2.99
NT01EI_1415	Bifunctional polymyxin resistance protein ArnA	-1.67	-1.51	-1.54	-2.31
**Purine metabolism and pyrimidine metabolism STRING clusters**					
NT01EI_3523	Selenium metabolism protein SsnA, putative	-1.60	-11.63	3.97	4.03
NT01EI_3529	Carbamate kinase	-2.10	-6.87	3.07	3.32
NT01EI_3530	Dihydropyrimidinase, putative	-2.58	-7.52	2.71	2.69
NT01EI_3531	M20/DapE family protein YgeY, putative	-2.11	-10.93	2.81	2.64
NT01EI_3532	Diaminopropionate ammonia-lyase, putative	-2.46	-11.71	2.64	2.36
NT01EI_3533	Carbamoyltransferase YgeW, putative	-2.22	-17.63	3.51	2.89
NT01EI_0684	Xanthine dehydrogenase, Fe-S subunit, putative	-2.11	-2.28	2.53	2.23
NT01EI_0686	Xanthine dehydrogenase, molybdenum-binding	-1.77	-8.06	3.16	3.27
