# Supplementary material for: Investigation mechanisms of action and resistance of Edwardsiella ictaluri to trans-cinnamaldehyde
Source: PLoS One. 2026 Jan 7;21(1):e0340053. doi: 10.1371/journal.pone.0340053 (PMC12779148; doi:10.1371/journal.pone.0340053)
Supplement: S8 Table — (PDF) [file pone.0340053.s008.pdf]

S8 Table. Identified SNPs and indels in the D30-TC strain compared to the D30-BHI control.

Position	Type	Reference	Alternative	Product
**Carbohydrate and energy metabolism**				
2274971	ins	AGGGGGGAT	AGGGGGGGAT	2-deoxy-D-gluconate 3-dehydrogenase%2C N-ter
1248927	snp	A	G	2-oxoglutarate dehydrogenase E1 component
3304013	ins	GAG	GAAG	3-isopropylmalate dehydratase large subunit
3332483	snp	G	A	4-hydroxythreonine-4-phosphate dehydrogenase PdxA
2140834	ins	GAAAAACAG	GAAAAAACAG	Bifunctional glucose-1-phosphatase/inositol phosphatase
2868621	snp	G	T	Bifunctional hydroxymethylpyrimidine kinase/phosphomethylpyrimidine kinase
1446263	ins	ACCTCTCTT	ACCCTCTCTT	NADH-quinone oxidoreductase subunit NuoN
103694	snp	T	C	UDP-N-acetylglucosamine 2-epimerase
2669284	del	TCCCCCCGGCGT	TCCCCCGGCGT	Nicotinate phosphoribosyltransferase
220864	snp	C	T	F0F1 ATP synthase subunit A
439219	snp	T	C	glycogen debranching protein GlgX
2719081	ins	GAG	GAAG	Glycosyltransferase involved in LPS biosynthesis GR25 family
110212	snp	A	G	Guanosine-5'-triphosphate-diphosphate diphosphatase
3585606	snp	G	A	Peptidylprolyl isomerase
3486402	ins	GAC	GAAC	Phosphate transport regulator
2831846	del	TGGGGGGGGAGAC	TGGGGGGGAGAC	Phosphoribosylformylglycinamidine cyclo-ligase
2800281	ins	GAG	GAAG	Quinol dehydrogenase ferredoxin subunit NapH
401057	snp	G	A	Sugar/pyridoxal phosphate phosphatase YigL
3535732	complex	ACACG	ACCACC	Acetyltransferase
448498	ins	GAC	GAAC	Maltodextrin phosphorylase
**Ribosomal function**				
27237	snp	C	T	50S ribosomal protein L11
3620254	snp	T	C	Elongation factor P
**Amino acid metabolism**				
744174	del	ATTTTTTTTTCATCG	ATTTTTTTTCATCG	Agmatinase
3026532	snp	A	G	Aspartate racemase
901162	del	TAAAAAAGAAAAAGGC	TAAAAAGAAAAAGGC	Glutamate--cysteine ligase
767626	snp	G	A	Glycine dehydrogenase
1467602	snp	A	G	O-succinylbenzoate--CoA ligase
**Cell division and chromosome partitioning**				
3277293	snp	C	T	Cell division protein FtsA
824704	snp	T	C	Chondroitinase
1676603	snp	A	G	Chromosome partition protein MukF
2231164	ins	CGCGGCTGCGGCTGCGGCTGCGGCTGCGGCTGCGG	CGCGGCTGCGGCTGCGGCTGCGGCTGCGGCTGCGGCTGCGG	Cobyric acid synthase
1894871	del	GTTCTG	GTCTG	Decarboxylating cobalt-precorrin-6B (C(15))-methyltransferase

255349	snp	G	A	Der GTPase-activating protein YihI
1403482	snp	A	G	Dihydroneopterin triphosphate 2'-epimerase
**DNA replication, repair, and modification**				
1742571	snp	G	A	DNA polymerase III subunit delta'
189311	snp	G	A	DNA topoisomerase (ATP-hydrolyzing) subunit B
3685248	snp	T	C	DNA topoisomerase III
23471	snp	T	C	DNA-directed RNA polymerase beta subunit
568538	snp	G	A	Excinuclease ABC subunit UvrA
2745425	snp	G	A	Exodeoxyribonuclease I
1196310	snp	C	T	tRNA (N6-isopentenyl adenosine(37)-C2)-methylthiotransferase MiaB
196537	del	CTTTTTTCTTGC	CTTTTTCTTGC	tRNA uridine-5-carboxymethylaminomethyl(34) synthesis GTPase MnmE
1507860	snp	C	T	Nucleoid-associated protein YejK
3595185	snp	G	A	Adenylosuccinate synthase
**Envelope stress**				
3148691	snp	T	C	Envelope stress response activation lipoprotein NlpE
1742571	snp	G	A	Metal-dependent hydrolase
1947097	snp	G	A	Methylmalonate-semialdehyde dehydrogenase (CoA acylating)
**Cell Envelope, Membrane Proteins, and Transport**				
352628	snp	G	A	MFS transporter family glucose-6-phosphate receptor UhpC
1189734	snp	T	C	Apolipoprotein N-acyltransferase
290555	snp	A	C	Porin
64809	snp	G	A	Omp85 domain-containing protein
3710874	ins	GTC	GTTC	TMAO reductase system periplasmic protein TorT
3199771	snp	T	G	N-acetylmuramoyl-L-alanine amidase AmiC
3021537	snp	C	T	N-acetylneuraminate lyase
2373996	snp	T	C	Sodium-independent anion transporter
746254	snp	C	T	Sulfatase
424599	snp	C	A	Amino acid ABC transporter substrate-binding protein
**Transcription and transcriptional regulation**				
2239229	snp	C	T	Transcriptional regulator TyrR
3061542	ins	AGGCGCGGCGCGGCA	AGGCGCGGCGCGGCGCGGCA	Two component system response regulator
3062409	snp	A	G	Two component system sensor kinase
3062964	snp	T	G	Two component system sensor kinase
1785262	snp	G	A	Two-component system response regulator PhoP
238249	del	GTTCG	GTCG	Ribose operon transcriptional repressor RbsR
**Hypothetical and uncharacterized**				
1804843	del	CTTTTTTTCTTGA	CTTTTTTCTTGA	Hypothetical protein
2286687	snp	A	G	LPD38 domain-containing protein
2021081	snp	C	T	Hypothetical protein
545730	snp	G	A	Uncharacterized protein YrdD
1297464	del	TGGGGGGAC	TGGGGGAC	DUF808 domain-containing protein
2083329	snp	G	A	Phage-related tail fiber protein
2462717	snp	A	G	Protein of unknown function DUF72
1060076	snp	A	G	Large exoprotein involved in heme utilization or adhesion
