# Supplementary material for: Investigation mechanisms of action and resistance of Edwardsiella ictaluri to trans-cinnamaldehyde
Source: PLoS One. 2026 Jan 7;21(1):e0340053. doi: 10.1371/journal.pone.0340053 (PMC12779148; doi:10.1371/journal.pone.0340053)
Supplement: S9 Table — (PDF) [file pone.0340053.s009.pdf]

S9 Table. Identified alteration in the D60-TC strain compared to the D60-BHI control.

Position	Type	Reference	Alternative	Product
**Cell wall, envelope, and transport**				
2387361	snp	G	T	Anhydro-N-acetylmuramic acid kinase
3193202	snp	G	A	Apolipoprotein N-acyltransferase
2430725	ins	TCCCCCCCG CCTT	TCCCCCC CCGCCTT	D-ribose-binding periplasmic protein
323082	snp	A	G	Ribose ABC transporter substrate-binding protein RbsB
3549934	snp	A	G	Sulfatase
3636681	snp	A	G	Sulfatase
671727	del	GAACG	GACG	TMAO reductase system periplasmic protein TorT
806820	snp	A	G	Translocation and assembly module TamB
3094124	snp	G	A	Dethiobiotin synthase
2249243	snp	C	T	Dihydromonapterin reductase
1182835	snp	A	C	N-acetylmuramoyl-L-alanine amidase AmiC
**Central carbohydrate metabolism and energy**				
2242094	del	GTTTTTTCA	GTTTTTCA	Bifunctional glucose-1-phosphatase/inositol phosphatase
1514034	snp	C	A	Bifunctional hydroxymethylpyrimidine kinase/phosphomethylpyrimidine kinase
1603342	ins	GAC	GAAC	Cysteine synthase A
2760145	snp	T	C	Cysteine/glutathione ABC transporter ATP-binding protein/permease CydC
338192	snp	A	G	F0F1 ATP synthase subunit A
3740917	snp	G	A	Glycerophosphodiester phosphodiesterase
3615309	snp	T	C	Glycine dehydrogenase (decarboxylating)
119847	snp	G	A	Glycogen debranching protein GlgX
1294783	snp	G	A	Guanine deaminase
3530382	snp	G	A	Hydrogenase 3 large subunit
3668996	snp	T	C	Hydrogenase-1 operon protein HyaE
1396434	snp	A	G	Maltodextrin glucosidase
110176	del	GAACG	GACG	Maltodextrin phosphorylase
1754786	snp	T	C	Methylglyoxal synthase
815133	snp	G	A	Octaprenyl diphosphate synthase
**Transcriptional regulation**				
3525203	snp	T	C	LacI family transcriptional regulator
1520869	snp	G	A	LysR family transcriptional regulator
320805	snp	A	G	Ribose operon transcriptional repressor RbsR
1438028	del	CTTTTTTAT	CTTTTTAT	Transcriptional regulator BolA
2597668	snp	C	T	Two-component system response regulator PhoP
**DNA replication, repair and partitioning**				
3716430	snp	A	C	Arm-DNA-bind-3 domain-containing protein
2763816	snp	G	C	ATP-dependent Clp protease ATP-binding subunit ClpA
2706327	snp	T	C	chromosome partition protein MukF
569744	snp	C	T	DNA topoisomerase 4 subunit B
1289	del	AGGGGGGG	AGGGGGG	DDE-Tnp-1 domain-containing protein
2564292	snp	T	C	DDE-Tnp-1 domain-containing protein
4069	del	AGGGGGGG	AGGGGGG	NEL domain-containing protein

**Antimicrobial resistance and stress response**				
1533130	snp	C	T	MdtA/MuxA family multidrug efflux RND transporter periplasmic adaptor subunit
1572455	snp	A	G	Multidrug efflux RND transporter permease AcrD
327908	snp	G	A	Low affinity potassium transporter Kup
896201	del	GTTCG	GTCG	Phosphate transport regulator
2137214	snp	C	T	Phage shock protein A
**Ribosomal function**				
2828078	snp	A	G	30S ribosomal protein S12 methylthiotransferase RimO
**Hypothetical and uncharacterized**				
1321055	del	CGCCGCGC CTCAGACG	CG	Hypothetical protein
1861339	snp	G	A	Hypothetical protein
2339311	snp	G	A	Hypothetical protein
2809674	snp	T	C	Hypothetical protein
2096240	snp	C	T	LPD38 domain-containing protein
